# Bayesian analysis of phase data in EEG and MEG

**DOI:** 10.7554/eLife.84602

**Published:** 2023-09-12

**Authors:** Sydney Dimmock, Cian O'Donnell, Conor Houghton

**Affiliations:** 1 Faculty of Engineering, University of Bristol Bristol United Kingdom; 2 School of Computing, Engineering & Intelligent Systems, Ulster University Derry/Londonderry United Kingdom; Max Planck Institute for Psycholinguistics Netherlands; University of Pennsylvania United States

**Keywords:** EEG, MEG, Bayesian, circular statistics, neurolinguistics, frequency-tagging, Human

## Abstract

Electroencephalography and magnetoencephalography recordings are non-invasive and temporally precise, making them invaluable tools in the investigation of neural responses in humans. However, these recordings are noisy, both because the neuronal electrodynamics involved produces a muffled signal and because the neuronal processes of interest compete with numerous other processes, from blinking to day-dreaming. One fruitful response to this noisiness has been to use stimuli with a specific frequency and to look for the signal of interest in the response at that frequency. Typically this signal involves measuring the coherence of response phase: here, a Bayesian approach to measuring phase coherence is described. This Bayesian approach is illustrated using two examples from neurolinguistics and its properties are explored using simulated data. We suggest that the Bayesian approach is more descriptive than traditional statistical approaches because it provides an explicit, interpretable generative model of how the data arises. It is also more data-efficient: it detects stimulus-related differences for smaller participant numbers than the standard approach.

## eLife digest

Phase coherence is a measurement of waves, for example, brain waves, which quantifies the similarity of their oscillatory behaviour at a fixed frequency. That is, while the waves may vibrate the same number of times per minute, the relative timing of the waves with respect to each other may be different (incoherent) or similar (coherent).

In neuroscience, scientists study phase coherence in brain waves to understand how the brain responds to external stimuli, for example if they occur at a fixed frequency during an experiment. To do this, phase coherence is usually quantified with a statistic known as ‘inter-trial phase coherence’ (ITPC). When ITPC equals one, the waves are perfectly coherent, that is, there is no shift between the two waves and the peaks and troughs occur at exactly the same time. When ITPC equals zero, the waves are shifted from each other in an entirely random way.

Phase coherence can also be modelled on phase angles – which describe the shift in each wave relative to a reference angle of zero – and wrapped distributions. Wrapped distributions are probability distributions over phase angles that express their relative likelihood. Wrapped distributions have statistics, including a mean and a variance. The variance of a wrapped distribution can be used to model phase coherence because it explicitly represents the similarity of phase angles relative to the mean: larger variance means less coherence.

While the ITPC is a popular method for analysing phase coherence, it is a so-called ‘summary statistic’. Analyses using the ITPC discard useful information in the trial-to-trial-level data, which might not be lost using phase angles.

Thus, Dimmock, O’Donnell and Houghton set out to determine whether they could create a model of phase coherence that works directly on phase angles (rather than on the ITPC) and yields better results than existing methods.

Dimmock, O’Donnell and Houghton compare their model to the ITPC using both experimental and simulated data. The comparison demonstrates that their model can detect entrainment of the brain to grammatical phrases compared to ungrammatical ones at smaller sample sizes than ITPC, and with fewer false positives. Traditional tools for studying how the brain processes language often yield a lot of noise in the data, which makes it difficult to analyse measurements. Dimmock, O’Donnell and Houghton demonstrates that the brain is not simply responding to the ‘surprise factor’ of words in a phrase, as some have suggested, but also to their grammatical category.

These results of this study will benefit scientists who analyse phase coherence. By using the model in addition to other approaches to study phase coherence, researchers can provide a different perspective on their results and potentially identify new features in their data. This will be particularly powerful in studies with small sample sizes, such as pilot studies where maximising the use of data is important.

## Introduction

In an electroencephalography (EEG) or magnetoencephalography (MEG) *frequency-tagged* experiment, the stimuli are presented at a specific frequency and the neural response is quantified at that frequency. This provides a more robust response than the typical event-related potential (ERP) paradigm because the response the brain makes to the stimuli occurs at the predefined stimulus frequency while noise from other frequencies, which will correspond to other cognitive and neurological processes, does not contaminate the response of interest. This quantification is often approached by calculating the inter-trial phase coherence (ITPC). Indeed, estimating coherence is an important methodological tool in EEG and MEG research and is used to answer a wide variety of scientific questions. There is, however, scope for improving how the phase coherence is measured by building a Bayesian approach to estimation. This is a per-item analysis and gives a better description of uncertainty. In contrast, the ITPC discards information by averaging across trials. As a demonstration, both approaches are compared by applying them to two different frequency-tagged experimental datasets and through the use of simulated data.

Frequency tagging is a well-established tool in the study of vision, where it is often referred to as the steady-state visual-evoked potential (Regan, 1966). At first, it was predominately used to study low-level processing and attention (see Norcia et al., 2015 for a review). Latterly, though, it been used for more complex cognitive tasks, such as face recognition and discrimination (Alonso-Prieto et al., 2013; Farzin et al., 2012; Liu-Shuang et al., 2014), perception of number (Guillaume et al., 2018; Van Rinsveld et al., 2020), and the ‘human quality’ of dance movements (Alp et al., 2017). It has been applied to other modalities: audition (Galambos et al., 1981; Picton et al., 2003; Bharadwaj et al., 2014), somatosensation (Tobimatsu et al., 1999; Galloway, 1990), and nociception (Colon et al., 2012; Colon et al., 2014). It has been used to study broad phenomenon like memory (Lewis et al., 2018) and lateralisation (Lochy et al., 2015; Lochy et al., 2016) along with more specific types of neurocognitive response, such as visual acuity (Barzegaran and Norcia, 2020) and the perception of music (Nozaradan, 2014). Furthermore, frequency tagging can be used in the assessment of disorders such as autism (Vettori et al., 2020a; Vettori et al., 2020b) and schizophrenia (Clementz et al., 2008). It has even been used to study neural responses to social interaction (Oomen et al., 2022). Beyond EEG and MEG, phase coherence has been proposed as a mechanism for signal routing (Abeles, 1982; Salinas and Sejnowski, 2001; Börgers and Kopell, 2008), assembly formation (Singer, 1999; Buzsáki, 2010), and coding (O’Keefe and Recce, 1993; Panzeri et al., 2010), so the measurement of phase coherence for electrocorticography, local field potentials, and neuronal spiking is important for the neuroscience of neuronal systems.

One striking application of frequency tagging is in neurolinguistics (Ding et al., 2016; Ding et al., 2017). Neurolinguistic experiments are difficult; since language experiments inevitably involve humans and target a phenomenon whose temporal grain is often too fine for magnetic resonance imaging, the principal neural imaging techniques are EEG and MEG. However, the complexity of the neural processing of language makes the signals recorded in these experiments particularly noisy, difficult to analyse, and difficult to disentangle from other cognitive processes. A further difficulty in neurolinguistics is that an ERP is often difficult to obtain because the tens or hundreds of repetitions required would render the stimulus meaningless to the participant, a phenomenon known as the semantic satiation (Jakobovits, 1962).

Consider, as an example, the frequency-tagged experiment described in Burroughs et al., 2021 and following the paradigm shown in Ding et al., 2017. This is used here as a paradigmatical example of a frequency-tagged experiment in neurolinguistics and, although the description here is specific to this example, much of the methodology is typical. In Burroughs et al., 2021, the neural response to phrases was investigated by comparing the response to grammatical adjective–noun (AN) phrases

$$...\underline{old\;rat}\;\underline{sad\;man\;ill}\;\underline{wife}...$$

to ungrammatical adjective–verb (AV) pairs

**Figure 1.**
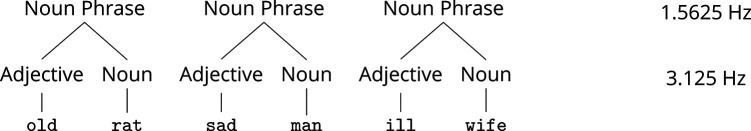
The syntactic target for the experiment. In the adjective–noun (AN) stimulus, there are noun phrases at the phrase rate, 1.5625 Hz; this structure is absent in the adjective–verb (AV) stimulus because AV pairs do not form a phrase. 3.125 Hz corresponds to the syllable rate in this experiment.

...rough give ill tell thin chew ...

where care had been taken to have a similar 2 g frequency for adjacent word pairs in each condition. The words are all presented at 3.125 Hz; however, the frequency of interest is the *phrase rate*, 1.5625 Hz, corresponding to the phrase structure of the AN stimuli (see Figure 1). In Burroughs et al., 2021, it is suggested that the strength of the response to AN stimuli relative to AV stimuli at this frequency measures a neural response to the grammatical structure, rather than to lexical category of the words.

This investigation required a quantitative measurement of the strength of the response. The obvious choice: the induced power at 1.5625 Hz does not work, empirically this proves too noisy a quantity for the stimulus-dependent signal to be easily detected and, indeed, although the frequency tag produces a more robust signal than an ERP, for more high-level or cognitive tasks, particularly neurolinguistic tasks, where frequency-tagging is now proving valuable, the power is not a useful measure; more needs to be done to remove the noise. Typically this is done by assuming the response is phase-locked to the stimulus, and so for frequency-tagged data in cognitive tasks it is common to use the ITPC. The ITPC is defined using the mean phase angle:

(1) $$\text{R}(f,\phi )=\frac{1}{K}\sum_{k}e^{i\theta_{fk\phi }}$$

where f is the frequency, k is the trial index, K is the number of trials, ϕ represents other indices such as electrode number or experimental condition, and $\theta_{f\,k\,\phi }$ is the phase of the complex Fourier coefficient for the EEG or MEG trace (f,k,ϕ). Across different applications, the mean phase angle is often called the mean resultant, a term we will use here. The ITPC is the length of the mean resultant:

(2) R(f,ϕ)=|R(f,ϕ)|

The ITPC is chosen as a quantitative measure to extract the portion of the response at the frequency of interest that is phase-locked to the stimulus and therefore consistent in phase from trial to trial. This is another of the denoising strategies required by these noisy data.

In the case of the experiment, we are discussing here the hold-all index ϕ is made up of participant, condition, and electrode indices. To produce a result, the ITPC is averaged over the 32 electrodes used in the experiment to give R⁢(f,p,c), where p labels participants, c labels conditions, and f labels frequency. The principal result of Burroughs et al., 2021 is that, in the language of frequentist statistics, R⁢(f=1.5626⁢ Hz,p,c=AN) is significantly larger than R⁢(f=1.5626⁢ Hz,p,c=AV).

**Figure 2.**
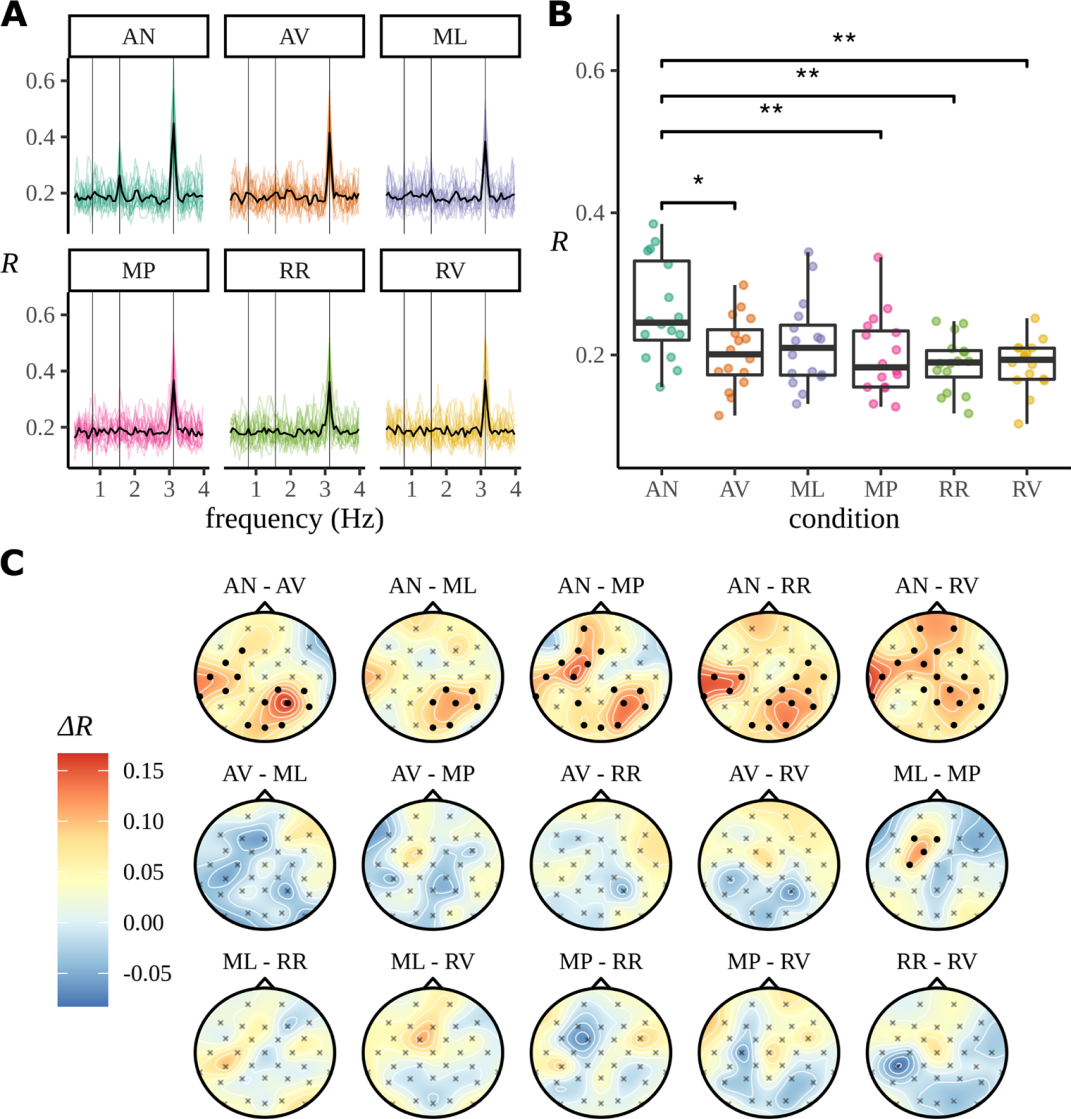
Summarising the inter-trial phase coherence (ITPC) for different conditions. (**A**) Coloured lines show the ITPC for each participant after averaging over electrodes and is traced across all frequencies. The mean trace, calculated by averaging over all participant traces, is overlaid in black. Vertical lines mark the *sentence*, *phrase*, and *syllable* frequencies as increasing frequencies, respectively. (**B**) Statistical significance was observed with an uncorrected paired two-sided Wilcoxon signed-rank test (∗0.05, ∗∗0.01). (**C**) ITPC differences for each condition pair calculated at the phrase frequency and interpolated across the skull. Filled circular points mark clusters of electrodes that were discovered to be significantly different (p<0.05) by the cluster-based permutation test.

The result of these experiments analysed using ITPC are summarised in Figure 2. This plots the ITPC measure for all six experimental conditions, the two, AN and AV, that have already been described and four others; a table of the experimental conditions is provided in Appendix 3. In Figure 2A, it is seen that there is a strong peak in ITPC at the syllable rate, 3.125 Hz, and, in the case of AN, at the phrase rate 1.5625 Hz. The ITPC at the phase rate is graphed in Figure 2B, where, again, it appears only AN has a response at the phrase rate.

In Burroughs et al., 2021, all analyses are done for ITPC averaged across electrodes; nonetheless in Figure 2C, we show the condition-to-condition difference in ITPC at each electrode, but averaged across participants:

(3) $$\Delta R_{ce}=\langle \Delta R_{pce}\rangle_{p}$$

where

(4) $$\Delta R_{pce}=R(f=1.5626\text{ Hz},e,c=\text{AN})-R(f=1.5626\text{ Hz},e,c=\text{RR}).$$

Visually these data appear to show a left temporal and right parietal response. However, the data are noisy and deciding the significance of any comparison is complicated: a straightforward calculation of the p-value using an uncorrected paired two-sided Wilcoxon signed-rank test gives a value <0.05 for 54 of the 480 possible comparisons. This includes some comparisons that fit well with the overall picture, for example, when comparing AN to AV the P4 electrode shows a difference with p=0.002 and the T7 electrode with p=0.044. It also includes some more surprising results: for the comparison of RR to RV, the CP5 electrode has p=0.0182 and the FC1 electrode has p=0.0213. If we interpret these as significant difference, the apparent difference between two conditions without an apparent phrase structure is odd and presumably misleading. However, a naïve Bonferroni correction would use a factor of 32×15, and in a manner typical of this very conservative approach, it massively reduces the number of significantly different responses, to one in this case.

As part of our reanalysis of these data, we used cluster-based permutation testing (Maris and Oostenveld, 2007) to identify significant clusters of electrodes for each ITPC condition difference, thereby providing a quantification of the observed structure in the headcaps. See Appendix 4 for an outline of the method. This statistical test is a widely adopted approach to significance testing EEG data because it does not fall prey to the high false-negative rate of a Bonferroni correction and takes advantage of spatiotemporal correlations in the data. However, this is different to testing individual electrode effects, and we must be careful to articulate this difference. With this method it is not possible to make inferential claims about the strength of the effect of any one electrode appearing in a significant cluster; electrodes appearing in significant clusters cannot be defined as significant as these comparisons are not controlled for under the null (Sassenhagen and Draschkow, 2019). As will be discussed later, this is a weaker claim than that based of the Bayesian posterior that can quantify this effect through a probabilistic statement.

There are a number of disadvantages to the ITPC. The most obvious problem is that the item in the statistical analysis of ITPC is a participant, not a trial. In the results described in Burroughs et al., 2021, the statistical significance relied on a t-test between conditions with a pair of data points for each participant: there are actually 24 trials for each participant but these are used to calculate the ITPC values. Some of the analysis in Burroughs et al., 2021 is done using 20 participants, some using 16; sticking to the latter for simplicity, the hypothesis testing is performed using 16 pairs of values, rather than 16×24=384 or even 16×24×32=12288 items if the individual electrodes are included. In short, the ITPC is itself a summary statistic, a circular version of variance, and so it hides the individual items inside a two-stage analysis

(5) items→ITPC→statistical analysis

However, this is hard to rectify: it is difficult to compare items across participants, or across electrodes, because the mean phase, phase[R(f,ϕ)], is very variable and not meaningful to the scientific questions of interest. This variability is graphed in Figure 4: this figure shows the value of

(6) $$\mu_{pe}=phase\;[\text{R}(f=1.5625,p,e,c=\text{AN})]$$

the phase of the mean resultant for the AN condition. For illustrative purposes, three example electrodes are picked out and the distribution across participants is plotted. What is clear is how variable these phases are; this means that individual responses cannot be compared across participants and electrode since p and e have such a strong effect on their value.

There are other classical tests of coherence which use phase information. One example is the Rayleigh test (Rayleigh, 1880; Rayleigh, 1919); this test can be used to check for either significant departure from uniformity or from the ‘expected phase’, that is, a particular phase angle specified by the researcher based on some other insight into the behaviour. Other tests, such as Hotelling’s $T^{2}$, apply jointly to phase and amplitude (Hotelling, 1931; Picton et al., 2001; Picton et al., 1987). These classical approaches are incompatible with the neurolinguistic study presented here. Firstly, it would be difficult to provide an expected phase; as demonstrated in Figure 4, the mean phase angle is highly variable across participants. There is also no substantive prior information available that could be used to supplement this value because language experiments vary from experiment to experiment. Secondly, because of the problem of semantic satiation the experiments we consider here are relatively short and lack the frequency resolution these classical approaches require.

Here we provide a Bayesian approach to phase data. We believe this has advantages when compared to the ITPC: it permits a per-item analysis and correspondingly a more statistically efficient and richer use of the data. Furthermore, as a Bayesian approach, it supports a better description of the data because it quantifies uncertainty and because it describes a putative abstraction of the stochastic process that may have generated the data while explicitly stating the underpinning assumptions. This replaces a hypothesis-testing and significance-based account with a narrative phrased in terms of models and their consequences, so, in place of an often contentious or Procrustean framework based on hypotheses, a Bayesian approach describes a putative model and quantifies the evidence for it.

A Bayesian account starts with a parameterised probabilistic model of the data. The model proposes a distribution for the data given a set of model parameters: this is the likelihood. In our case, the likelihood will be the probability distribution for the phases of the responses, given our model. Our interest is in how the variance of this distribution depends on the condition. In addition to the likelihood, the full Bayesian model also includes a prior distribution for parameters, for example, it includes priors for the parameters which determine the relationship between the condition and the distribution of phases. The goal is to calculate the *posterior distribution*, the probability distribution for the parameters given the experimental data; this follows from Bayes’ theorem:

(7) $$P(\Theta |\Delta )=\frac{P(\Delta |\Theta )P(\Theta )}{P(\Delta )}$$

where Θ are the parameters and Δ the data. Essentially, P⁢(Δ|Θ) is the likelihood, the distribution of the data given some parameters: the goal is to take the data and from them calculate the posterior distribution of the parameters: P⁢(Θ|Δ). The denominator P⁢(Δ) can usually be ignored because it is just a normalising constant that is independent of the parameter values and therefore does not change the shape of the posterior distribution. There are a number of excellent descriptions of the Bayesian approach to data, including the textbook (Gelman et al., 1995) and a recent review (van de Schoot et al., 2021); our terminology and notation will often follow conventions established by the textbook (McElreath, 2018).

In many ways Bayesian descriptions are more intuitive and easier to follow than the frequentist approaches that have been favoured over the last century. The impediment to their use has been the difficulty of calculating the posterior distribution. These days, however, powerful computing resources and new insight into how to treat these models mean that there are a variety of approaches to estimating the posterior; one approach, the one used here, is to sample from the posterior without calculating it analytically using Markov chain Monte Carlo (MCMC) techniques. Probabilistic programming languages such as Stan (Carpenter et al., 2017) and Turing (Ge et al., 2018) make it easy to use advanced MCMC sampling methods such as Hamiltonian/Hybrid Monte Carlo (HMC) and the no U-turn sampler (NUTS) (Duane et al., 1987; Neal, 2011; Betancourt, 2013), making the complexity of a frequentist analysis unnecessary. Here, we report results calculated using Stan though many of the computations were carried out in both Stan and Turing.

## Materials and methods

### Data

In this article, we consider two experimental datasets and simulated data. The first experimental dataset is the phrase data described above; this can be considered the primary example, and these data are familiar to us and formed the target data while developing the approach. In this section, the methods are described with reference to these particular data; we believe using a particular example allows us to describe the method with greater clarity. However, to demonstrate the generality of the approach we also apply it to another dataset measuring statistical learning of an artificial language. These data are described briefly here. The experiments we performed with simulated data used data generated by the Bayesian model with different effect sizes; this is described in the ‘Results’ section.

The second experimental dataset is related to statistical learning of an artificial language. Statistical learning refers to the implicit capacity of the brain to extract the rules and relationships between different stimuli in the environment. We used our Bayesian model to analyse data from an interesting frequency-tagged experiment that investigated statistical learning in an artificial language task (Pinto et al., 2022). In the experiment, 18 syllables are arranged into six three-syllable words and played in a stream so that the transition probabilities inside a pseudoword are 1 while the transition between the last syllable of a pseudoword and the first of another is 0.2. The goal is to assess the degree to which pseudowords are learned. A frequency-tagged paradigm was used. The syllables were presented at a constant rate f, such that three-syllable pseudowords had a frequency of f/3. Evidence of statistical learning can then be quantified using ITPC at this frequency and its harmonics.

**Figure 3.**
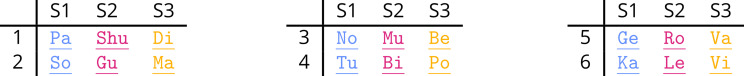
Pseudowords and position-controlled syllables. All six pseudowords used in the experiment are numbered. Groups of position-controlled syllables have been coloured.

In the experiment, syllables were presented at 4 Hz, resulting in a three-syllable pseudoword frequency of 1.33 Hz. Each participant was subject to two conditions, which we refer to as baseline (BL) and experimental (EXP) in line with Pinto et al., 2022. For the EXP condition, there were six pseudowords with a between-word transitional probability of 0.2; a summary of these are shown in Figure 3. The BL condition contained the same 18 syllables as EXP, but adopted different transitional probability rules to remove regularities. In this study, recordings were taken from 40 adult participants (25 females, 35 right-handed, ages 20–38) using 64 electrodes sampled over three blocks of 44 trials; of these participants, 39 had complete EEG recordings to use in the analysis. In the ‘Results’ section, we recapitulate the original ITPC analysis of these data and consider a Bayesian model.

### Circular distributions

Here, the data are a set of phases and so the model for the data is a probability distribution on a circle. The motivation which informs the ITPC is that the phases to a greater or lesser extent have a unimodal distribution around the circle and so the model should be a unimodal distribution on the circle (see Figure 5). One common class of distributions on the circle is given by the wrapped distributions with probability density function

(8) $$p(\theta )=\sum_{n=-\infty }^{\infty }p_{r}(\theta +2\pi n)$$

where $p_{r}\,\left(x\right)$ is a probability density of a distribution on the real line and θ is the angle. It might seem that the obvious choice for $p_{r}\,\left(x\right)$ would be the Gaussian distribution. In fact, the wrapped Gaussian distribution is not a very satisfactory example of a wrapped distribution because p⁢(θ) cannot be calculated in closed form. A much better example is the Cauchy distribution

(9) $$p_{r}(x)=\frac{1}{\pi \gamma }\frac{\gamma^{2}}{(x-x_{0}{)}^{2}+\gamma^{2}}$$

where *x*_0_ is a parameter giving the median of the distribution and γ is a scale parameter; the corresponding wrapped distribution has the closed form:

(10) $$p(\theta )=\frac{1}{2\pi }\frac{\sinh\gamma }{\cosh\gamma -\cos\left(\theta -\mu \right)}$$

and, in contrast to the Cauchy distribution on the real line, where the moments are not defined, the wrapped distribution has a well-defined and convenient value for the mean resultant:

(11) $$\text{R}=e^{i\mu -\gamma }$$

The circular variance S of the wrapped Cauchy distribution can be derived from the length of this complex vector:

(12) $$\begin{array}{rl} S & =1-|\text{R}| \end{array}$$

(13) $$\begin{array}{rl} & =1-e^{-\gamma } \end{array}$$

Thus, as illustrated in Figure 5A, a large value of γ corresponds to a highly dispersed distribution; a low value to a concentrated one. With this explicit relationship between parameter values and the mean resultant, the Cauchy distribution is a convenient choice for our model.

### Prior distributions

**Figure 4.**
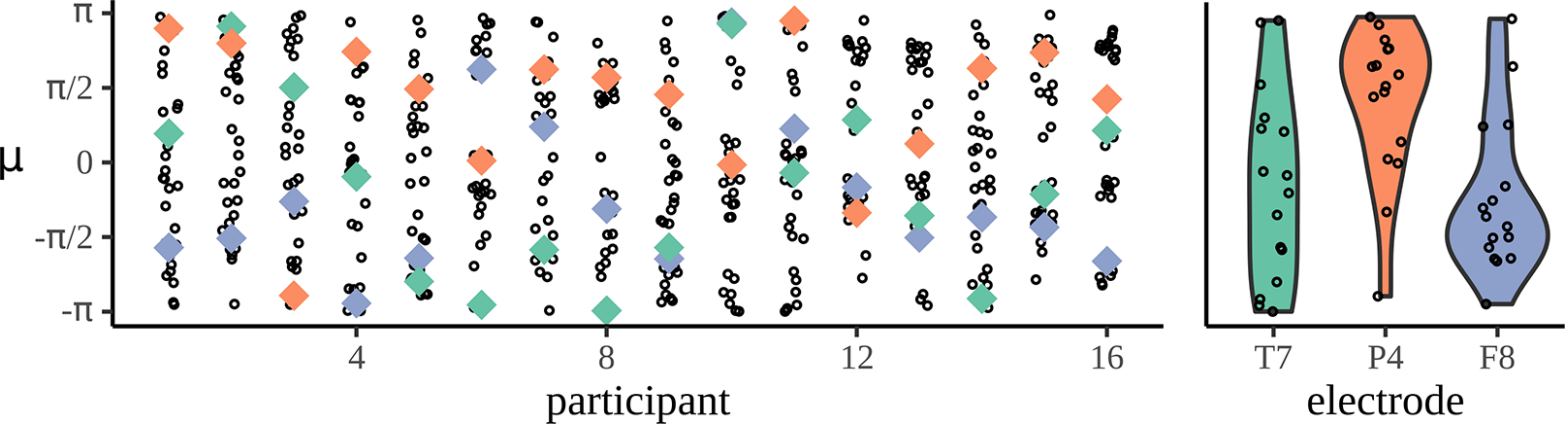
Mean phases are uniform across participants. The left-hand panel shows the distribution of phases across electrodes for each participant: each column corresponds to one participant and each dot marks the mean phase µ for each of the 32 electrodes calculated at the phrase frequency for the adjective–noun (AN) grammatical condition. To show how a given electrode varies across participant, three example electrodes are marked, T7 in green, P4 in orange, and F8 in purple. The right-hand panel shows the distribution of mean phases, µ, across participants.

The next important element is the choice of priors both for the mean of the wrapped distribution, µ, and for γ, which determines how dispersed the distribution is. The prior for µ is the more straightforward: a different value of µ is required for each participant, condition, and electrode. This prior should be uniform over the unit circle (Figure 4). Although there is likely to be correlations in µ values for the same electrode across participants and for the electrodes for a given participant, since the value of µ is not of interest, it is convenient to ignore this and pick an independent value $\mu_{p\,c\,e}$ for each triplet of participant–condition–electrode values. Future studies that aim to extend this model could consider adding correlations to µ.

Since µ has a uniform prior over the unit circle, it would seem that the correct prior is

(14) $$\mu_{epc}∼Uniform(-\pi ,\pi )$$

This is, however, wrong; the ideal distribution is a uniform distribution on the circle, not on the interval, and while the uniform distribution on an interval has the same numerical probability values, it has a different topology. This matters when sampling using an MCMC method. In MCMC, to create the list of samples, referred to as the chain, the sampler moves from sample to sample, exploring the parameter space. In this exploration for µ, if the posterior value is, for example, close to π, then the chain should explore the region near to π, which includes values near -π in [-π,π). A small change should move the sampler from π to -π. However, dynamics on the interval [-π,π) can only get from one to the other by traversing the potentially low-likelihood interval in between. Nothing in the mathematical description of the common MCMC samplers, such as NUTS, prevents the prior from being defined on a circle or other compact region. However, there is a problem: the current high-quality implementations of these methods in do not allow priors over circles.

### Sampling from a circular prior distribution

**Figure 5.**
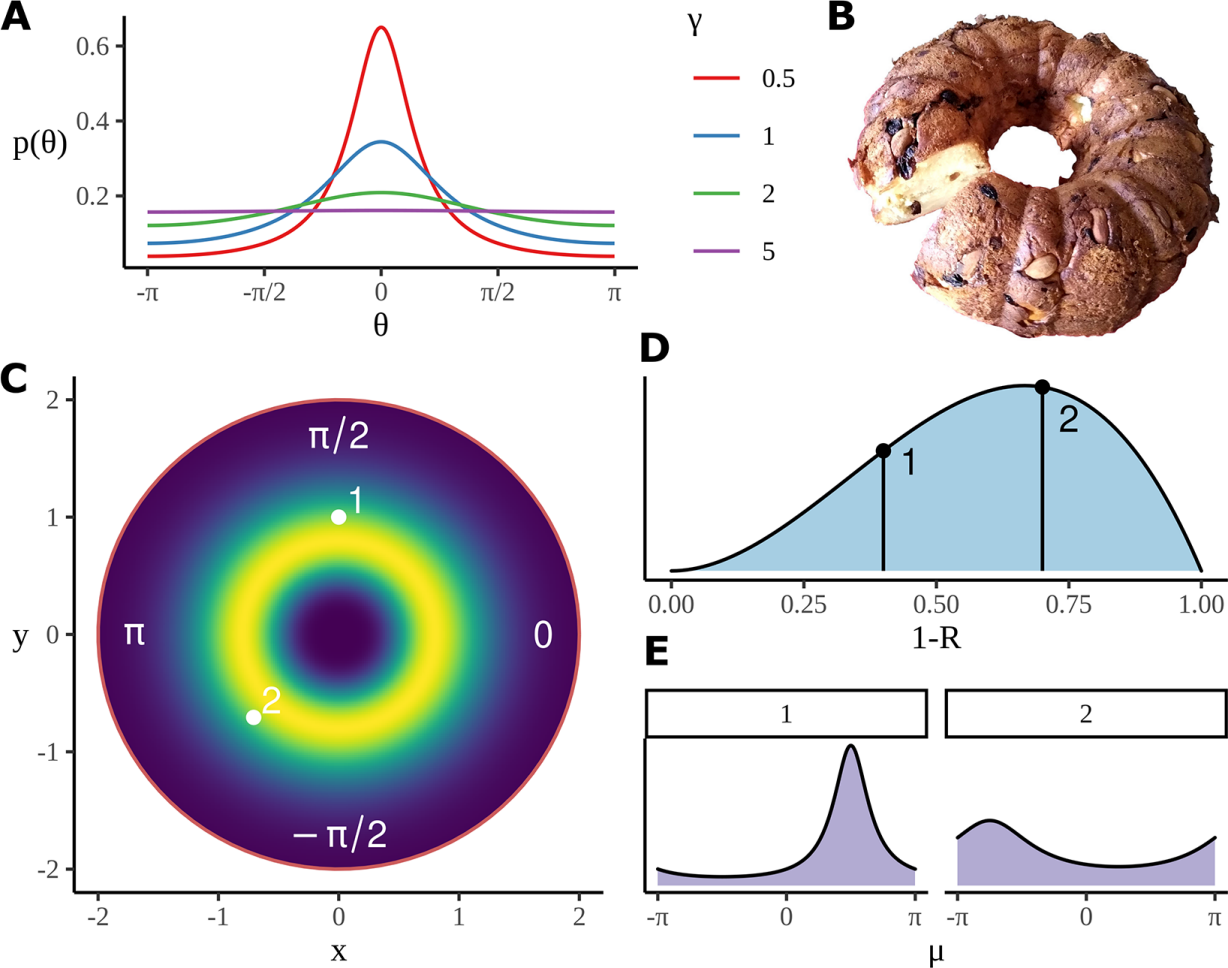
Model construction and geometry. (**A**) Example wrapped Cauchy density functions for different values of the scale parameter. (**B**) The Bundt distribution has a shape reminiscent of a cake made in a Bundt tin. (**C**) The mean phase is sampled from an axially symmetric prior distribution with a soft constraint on the radius. Highlighted pairs (x,y) give the location of example points; these points correspond to the mean angle for a wrapped Cauchy distribution using angle⁢(x,y). (**D**) An example distribution for S=1-R which determines the γ parameter in the wrapped Cauchy distribution. S is related to other parameters such a condition and participant number through a logistic regression, as in Equation 19, the priors for the slopes in the regression are used to produce the distribution shown here; as before, two example points are chosen, each will correspond to a different value of γ in the corresponding wrapped Cauchy distribution. (**E**) Example wrapped Cauchy distributions are plotted in correspondence with the numbered prior proposals in (**C**) and (**D**).

As a practical approach to avoiding this difficulty, we introduce a two-dimensional distribution which, in polar coordinates, is uniform in the angle coordinate and in the radial part restricts sampling to a ring around the origin. Because its probability density function resembles the Bundt cake tin, used to make kugelhopf (Hudgins, 2010, see Figure 5B), this will be referred to as a Bundt distribution. The choice of the radial profile of the Bundt distribution is not critical; its purpose is to restrict the samples to a ring: we sample points (x,y) on a plane so that their radius $\rho =\sqrt{x^{2}+y^{2}}$ is drawn from a gamma distribution

(15) ρ∼Gamma(10,0.1).

giving what we will call a Bundt-gamma distribution. This distribution has mean 1 and standard deviation 0.1, giving the golden ring of likely (x,y) values seen in Figure 5C. In fact, the radial values are not used in the model; what is used is the angle:

(16) $$\mu_{pce}=angle(x_{pce},y_{pce})$$

### A linear model for the scale of the wrapped Cauchy distribution

The final element of the model is the prior for γ; obviously the intention is to have this depend on the condition. To make our priors easier to interpret, it is convenient to use a link function, first converting from γ to the circular variance S:

(17) $$\gamma_{pce}=-\log(1-S_{pce})$$

S is bound between 0 and 1, so a second link function is applied

(18) $$S_{pce}=\sigma (\upsilon_{pce})$$

where σ⁢(υ) is the logistic function. The quantity $\upsilon_{p\,c\,e}$ quantifies the effect of participant, condition, and electrode on response. In this model it is linear

(19) $$\upsilon_{pce}=\alpha_{c}+\beta_{pc}+\delta_{ce}$$

so $\alpha_{c}$ is understood as quantifying the effect of condition, $\beta_{p\,c}$ the effect of the participant, and $\delta_{c\,e}$ the effect of electrode. In the language of regression, these are slopes. In the case of $\beta_{p\,c}$ and $\delta_{c\,e}$, experimenting with different models has demonstrated a better fit when these are interaction terms, allowing the effect of respectively participant and electrode to be condition dependent.

Thus, the main objects of interest are $\alpha_{c}$, $\beta_{p\,c}$, and $\delta_{c\,e}$, and our result is calculated by sampling the posterior distribution for these quantities. Of course, these quantities also require priors. The obvious place to start is the condition effects *a*_*c*_; because effects are weak in these data, our prior belief is that for any condition the circular variance should be reasonably large, likely bigger than a half. Conversely, the parameters $\beta_{p\,c}$ and $\delta_{c\,e}$ correspond to deviations about the baseline level $\alpha_{c}$ which can be represented easily using unbounded symmetric distributions. The prior for the slopes $\beta_{p\,c}$ has a hierarchical structure, allowing correlations across conditions; $\beta_{p\,c}$ models the participant response: roughly speaking the idea that a participant who is not paying attention in one condition is likely to be inattentive for all of them. The participants slopes, $\beta_{p\,c}$, were assigned a multivariate t-distribution, chosen because its heavy tails give a more robust estimation in the presence of ‘unusual’ participants: exceptionally strong or exceptionally weak, probably due to lack of attention. This multivariate parameterisation allows for a simultaneous two-way regularisation process due to information sharing both within conditions and across conditions. The idea of self-regularising priors is common in hierarchical Bayesian models and is often referred to as partial pooling (see Gelman et al., 1995 for a review). A similar approach was adopted for the electrode slopes, but with partially pooling only within condition, and not across conditions: testing showed that this was not useful. These priors are described in further detail as part of a full description of the model in the supporting information (see Appendix 1).

At the moment one disadvantage of Bayesian analysis is that the process of selecting priors is unfamiliar and this might appear intimidating, particularly for experimental scientists hoping to benefit from the approach without being interested in the nitty-gritty of defining priors. Hopefully, as our understanding matures, this process will become both better established and better understood, with good default choices available as suggestions from analysis libraries.

## Results

The posterior distribution was sampled using the NUTS algorithm implementation in Stan. Four chains were run for 4000 iterations, with half dedicated to a warm up or calibration epoch. Details of the software packages and libraries used can be found in Appendix 2.

### Comparison with ITPC results

**Figure 6.**
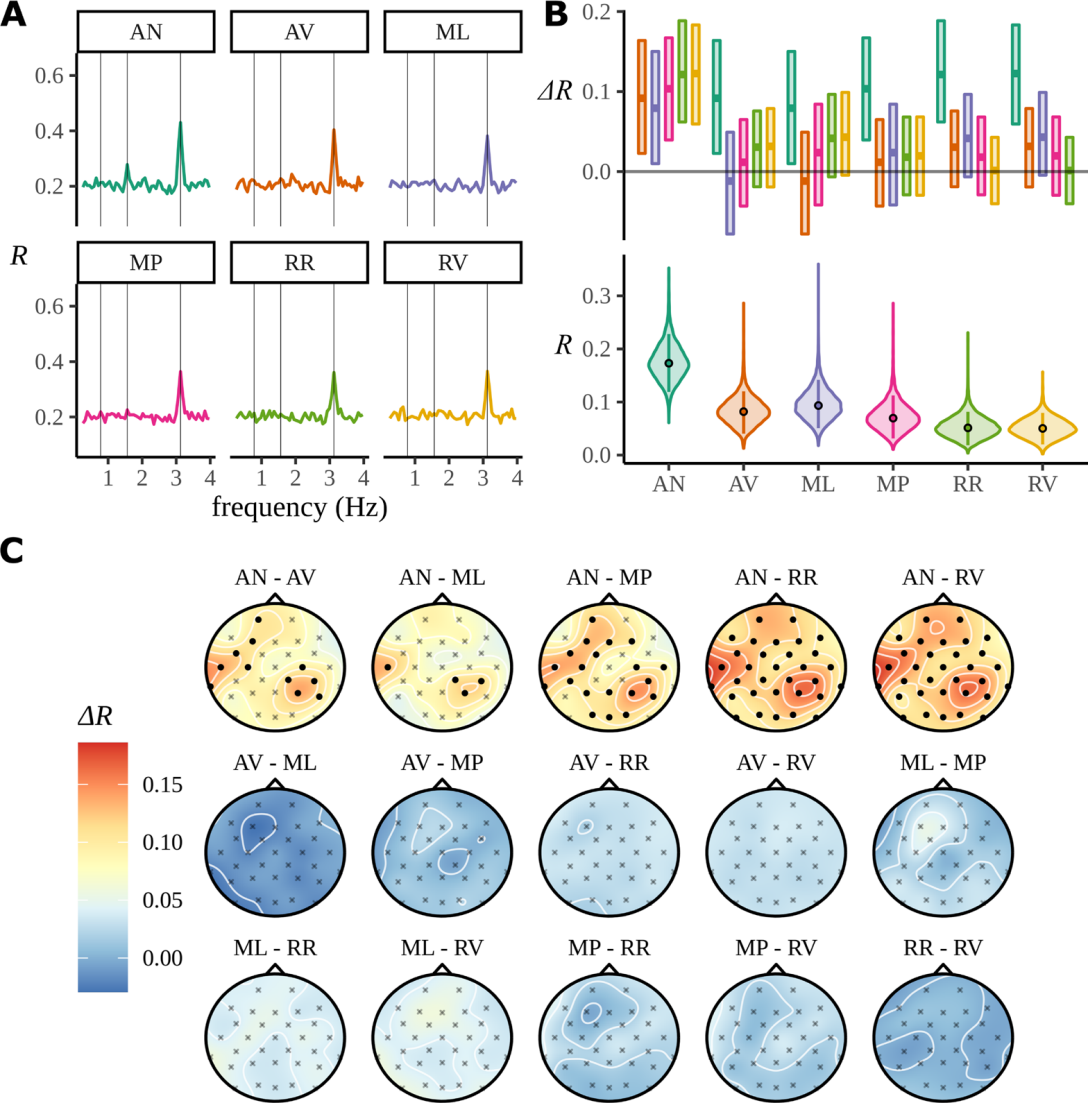
Posterior distributions. (**A**) The traces show point estimates of the mean resultant length calculated across all 58 frequencies using the optimisation procedure. (**B**) The marginal posterior distributions for each transformed condition effect $\alpha_{c}$ are shown with a violin plot. Posteriors over condition differences are given directly above, the colour of which represents the condition against which the comparison is made. For example, the green interval above the adjective–verb (AV) condition describes the posterior difference AN-AV. Posterior differences and marginal intervals are all given as 90% highest density intervals (HDIs) marked with posterior medians. (**C**) Posterior medians are interpolated across the skull for all condition comparisons. Filled circle shows those electrodes where zero was not present in the 95% HDI for the marginal posteriors over the quantity in Equation 22.

The posterior distributions are described in Figure 6. This figure reprises, using our analysis, the ITPC analysis exhibited in Figure 2. Figure 6A shows a point estimate of the mean resultant length across all frequencies estimated using the optimise function within RStan; as in the earlier figure (Figure 2A), there is a phase peak visible at the phrase frequency 1.5626 Hz for AN, but not for the other conditions.

Figure 6B represents our attempt to find a way to present the results of Bayesian analysis in a way which resembles as much as possible the ‘significance difference bracket’ often used in presenting experimental results. At the bottom of Figure 6B, we see the posterior distributions over the mean resultant length for each condition. These posteriors are obtained by transforming posterior samples of the parameter $\alpha_{c}$, which describes the effect of condition on the response within the regression to circular variance $S_{c}$, as described in Equation 18, then subtracting from one to obtain the mean resultant length.

(20) $$\begin{array}{rl} R_{c} & =1-S_{c} \end{array}$$

(21) $$\begin{array}{rl} & =1-\sigma (\alpha_{c}) \end{array}$$

It appears that the AN condition has a higher value of the mean resultant length than the other five conditions. To examine this further, the upper panel in Figure 6B also shows the 90% highest density intervals (HDIs) and posterior medians of the posterior distribution over the differences between the mean resultant length of all condition pairings. The HDI provides a summary of the full posterior distribution: it is the smallest width interval that contains a specified proportion of the total probability, and here, above the violin plot for each $\alpha_{c}$, we have plotted the HDI for that condition relative to the other four: this could be considered as a Bayesian equivalent to the confidence brackets common in frequentist plots like Figure 2. Here, only the HDIs that do not overlap zero are the ones corresponding to a difference between AN and another condition: this clearly shows that in our model there is a neural response at the phrase stimulus frequency for AN but not for the other conditions. It appears, for example, that although the MP condition consists of grammatical phrases, the fact that these phrases are of different types means that there does not appear to be a response. This suggests that the neuronal response observed for AN is a response to a specific type of phrase, not to any phrase.

In Figure 6C, we see the electrode-by-electrode comparisons across conditions. These graphs show a clearer structure than the corresponding ITPC analysis in Figure 2C; there is a left temporal and right parietal response for AN and nothing else. In an attempt to draw a comparison with the headcaps in Figure 2C, we have highlighted the electrodes whose marginal posteriors did not contain zero. This is not a one-to-one comparison: in Figure 2C, no claims of significance can be made about any specific electrode, only the clusters of activity themselves are significant. Here we are presenting evidence given by the Bayesian model based on each marginal distribution and argue that false-positives arising from these 32 comparisons should be reduced by the multivariate machinery of the Bayesian model. In Figure 6C, only a summary of the posterior for each electrode-by-electrode comparison is shown. It is important to note that, in contrast with the ITPC analysis in Figure 2C, the posterior is much more than a point estimate. In Appendix 9, an example of the plausible patterns of activity captured by the posterior distribution for the AN–AV comparison is provided.

### Participant effects

**Figure 7.**
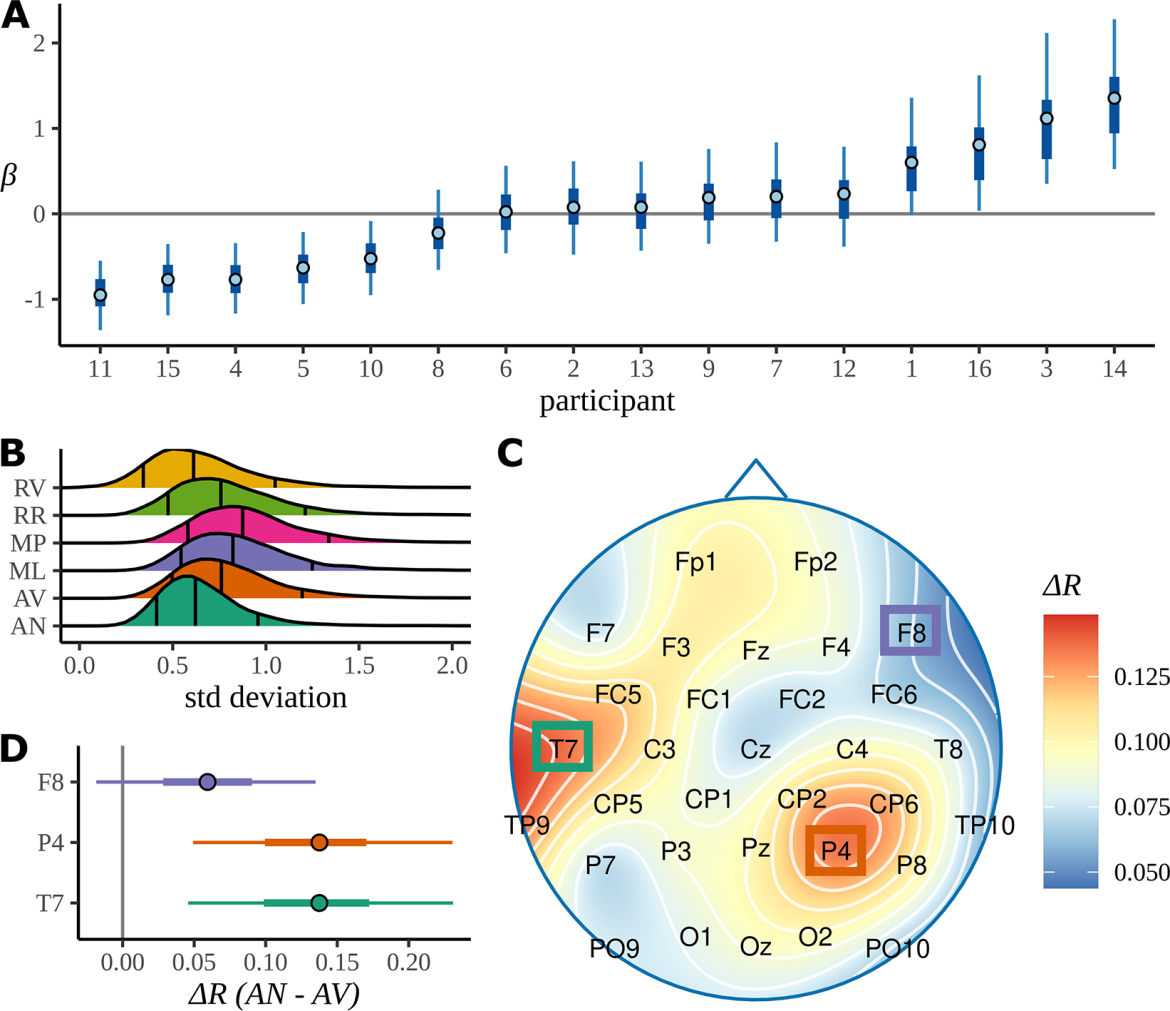
Participant attentiveness and localised electrode effects. (**A**) The intervals show participant effects for the grammatical adjective–noun (AN) condition given as 50/90% highest density intervals (HDIs) and posterior medians. (**B**) The posterior distributions over the standard deviation of participant slopes for each condition. Outer vertical lines mark the 90% posterior HDIs, inner lines mark the posterior median. (**C**) The skull plot from Figure 6C for the AN–AV difference with electrode names marked. (**D**) Posterior distributions over electrode differences for those positions on the skull where the grammatical condition shows a higher coherence of phases at the average participant in (**C**). Intervals give 50/90% HDIs and the posterior medians. AV, adjective–verb.

In Figure 7A, we plot the 90% HDIs for the participant slopes, $\beta_{p\,c}$, for c= AN; more positive values of β correspond to less attentive participants, more negative values correspond to more attentive. These have been arranged in increasing order of β with the participant number p given on the x-axis. From an experimental point of view, this plot gives some reassurance that there is no systematic trend, with participation becoming better or worse as the experiment progressed through participants. Our model includes a condition-dependent standard deviation for the participant response (see Appendix 1); posterior distributions for these standard deviations are plotted in Figure 7B. This appears to indicate that there is more across-participant variation in responses to the MP and ML conditions, where there is a structure but a complicated or confusing one, than to either the highly structured and grammatical AN condition or the RV and RR conditions, with little or no structure at the phrase rate.

### Electrode effects

To investigate the electrode-dependent response, Figure 7C is an enlarged version of the first headcap plot from Figure 6C: the difference in mean resultant between AN and AV. The heatmap colour scale is recalibrated since here it refers only to this one plot. The localisation of the response is seen very clearly. It is difficult to combine a headcap plot and information about the posterior distribution, so the HDI for

(22) $$\Delta R_{e}=R_{c_{1}e}-R_{c_{2}e}$$

where $c_{1}=AN$, $c_{2}=AV$ and

(23) $$R_{ce}=1-\sigma (\alpha_{c}+\delta_{ce})$$

is plotted for three example electrodes, one electrode from each of the two active areas and one from an area that shows little activity. The response for P4 and T7 is clearly different from zero, indicating that there is a stronger response to the AN condition than to the AV condition at these two electrode. The same HDI analysis for RR versus RN does not show any electrodes whose HDI does not overlap zero; the presumably misleading results for CP5 and FC1 noted in the discussion of ITPC results do not appear here.

**Figure 8.**
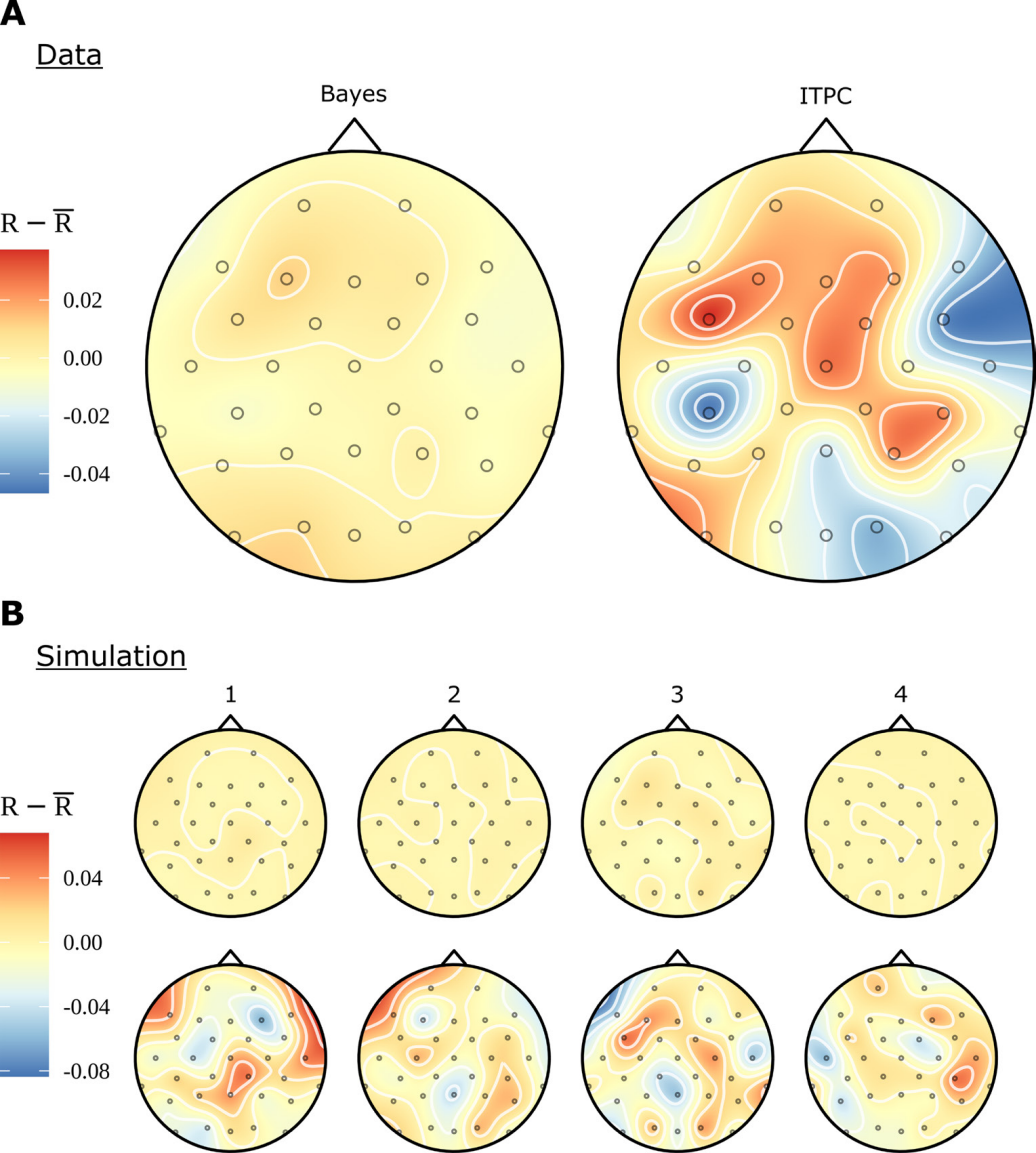
Comparison of electrode effects for no signal. (**A**) Topographic headcaps for the phrase data using the random words condition (RR). When calculating phase coherence using the inter-trial phase coherence (ITPC) for this condition, there is an apparent high but misleading variation in electrodes across the skull. This does not manifest in the Bayesian result due to regularisation of the electrode effects. (**B**) Data was simulated from the generative Bayesian model four times with electrode effects set to zero to provide a known ground truth. Plots 1-4 can be thought of as results from four separate experiments. On this simulated data, the ITPC shows variation similar to (**A**); the Bayesian results are consistent with the ground truth. The ITPC has an upward bias, so in all figures the mean was subtracted for ease of comparison.

In Figure 2C, we see that even for conditions, such as RR and RV, which contain no linguistic structure at the phase rate, there are patterns of electrode activity in the topographic headcaps. In contrast, the analogous Bayesian headcaps in Figure 6C did not show similar patterns. We used simulated data to investigate whether the Bayesian model is correctly demonstrating that there is no phrase-level response for these conditions, rather than the other possibility: that the beguiling patterns seen in the ITPC headcaps represent a real activity invisible to the Bayesian analysis. In fact, the evidence points to the first alternative; Figure 8 presents evidence that the Bayesian model is more faithful to the data when there is no meaningful variation in electrode effects. Figure 8A shows the real data again; however, whereas previously the headcap was plotted for differences between conditions, here we fit directly to the RR condition. There is no effect visible for the Bayesian headcap, but for the ITPC headcap there are variations that may suggest localised activity, even though this condition does not have any structure at the phrase rate. In Figure 8B, four datasets were simulated from the generative Bayesian model with electrode effects set to zero; other parameters were centred on the posterior means of the ungrammatical AV condition. The four simulations are marked as 1-4 in the figure. For simplicity, there is only one condition, but in other respects the simulated data mimics the real data: it has 16 participants, 32 electrodes, and 24 trials. These simulations are intended to represent four different iterations of the same experiment; apart from differing in any random numbers, they are identical. The data resulting from these four simulations were fitted with both methods. Evidently, the Bayesian results are much closer to the ground truth. The ITPC results show variations that could easily be misinterpreted.

### Sampler diagnostics

**Figure 9.**
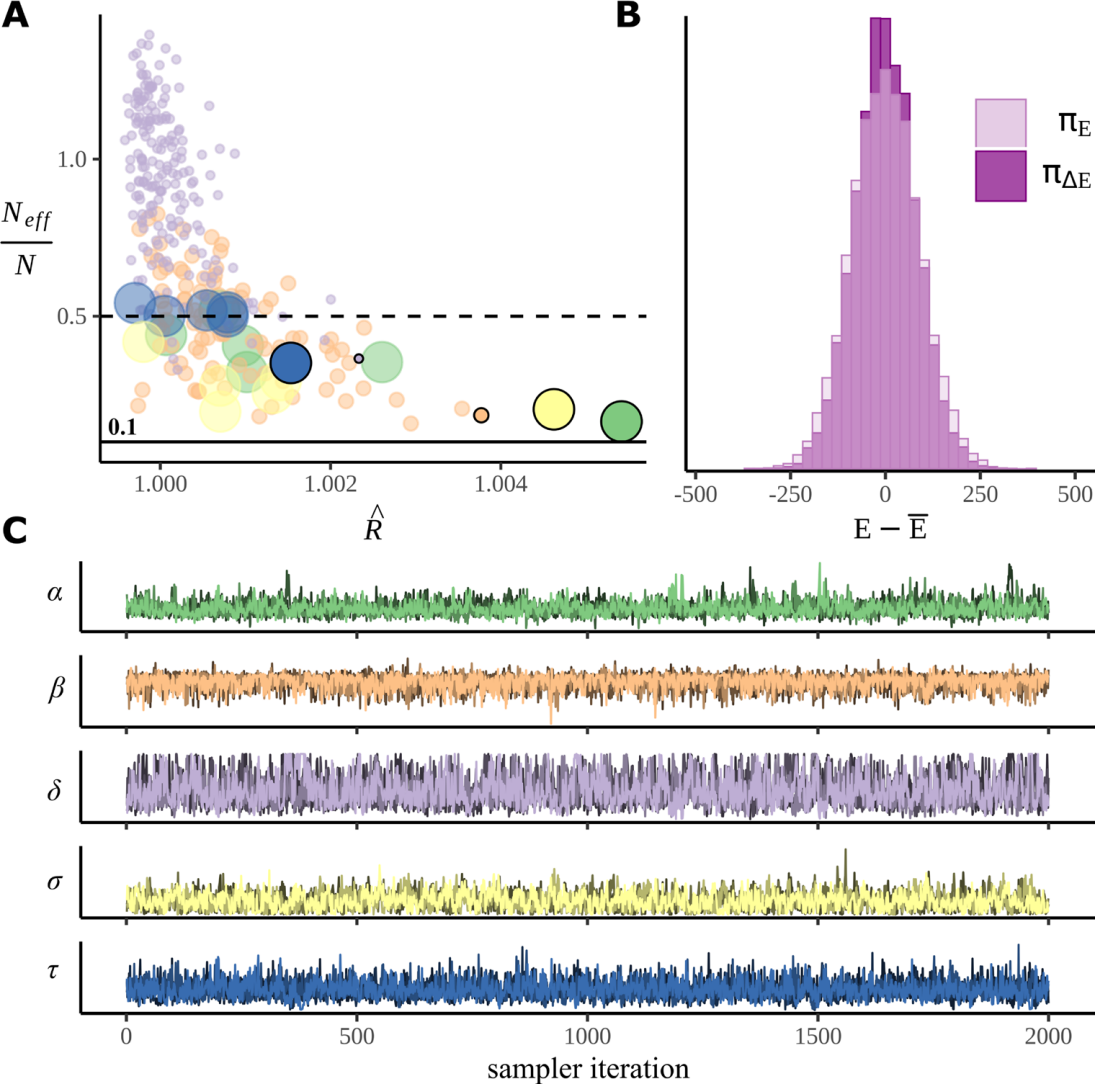
Sampler performance and diagnostics. (**A**) The performance of the sampler is illustrated by plotting $\hat{R}$ (R-hat) against the ratio of the effective number of samples for each parameter in the model. Points represent individual model parameters grouped by colour with a different colour for each parameter type. For convenience, the dot sizes are scaled, so the more numerous parameters have smaller dots, the less numerous, fewer, so, for example, $\alpha_{c}$ with only six examples, is large. (**B**) A histogram comparing the marginal energy distribution $\pi_{E}$, and the transitional energy distribution $\pi_{\Delta \,E}$ of the Hamiltonian. (**C**) Post-warmup trace plots. All four chains for the poorest performing parameter within each parameter group are overlaid. Corresponding points in (**A**) are marked with a black border and zero transparency.

When calculating posteriors using MCMC, it is necessary to check the success of sampling; sometimes it can become stuck in one part of the parameter space (Gelman et al., 1995; McElreath, 2018). Figure 9 plots the standard MCMC diagnostic measures calculated from our posterior samples. There does not appear to have been any problems: the most commonly used measure of the success of sampling is $\hat{R}$, often referred to as R-hat. This is a measure of convergence that compares the means and variances of chains; ideally it would be 1.0, but typically a value of <1.05 is considered acceptable and <1.02 desirable. Here, all values of R-hat are <1.006, indicating good mixing; values are plotted in Figure 9A; Figure 9C plots the chains for the parameter with the largest R-hat value for each parameter type; none of these plots appear to show the sort of pathological behaviour associated with poor sampling, and chains are both stationary and convergent. Another measure of sampling success, the comparison of marginal and transitional probabilities of the Hamiltonian, is exhibited in Figure 9B; this also indicates good sampling. See Appendix 2 for a note on tree depth warnings.

### Case study: Statistical learning for an artificial language

**Figure 10.**
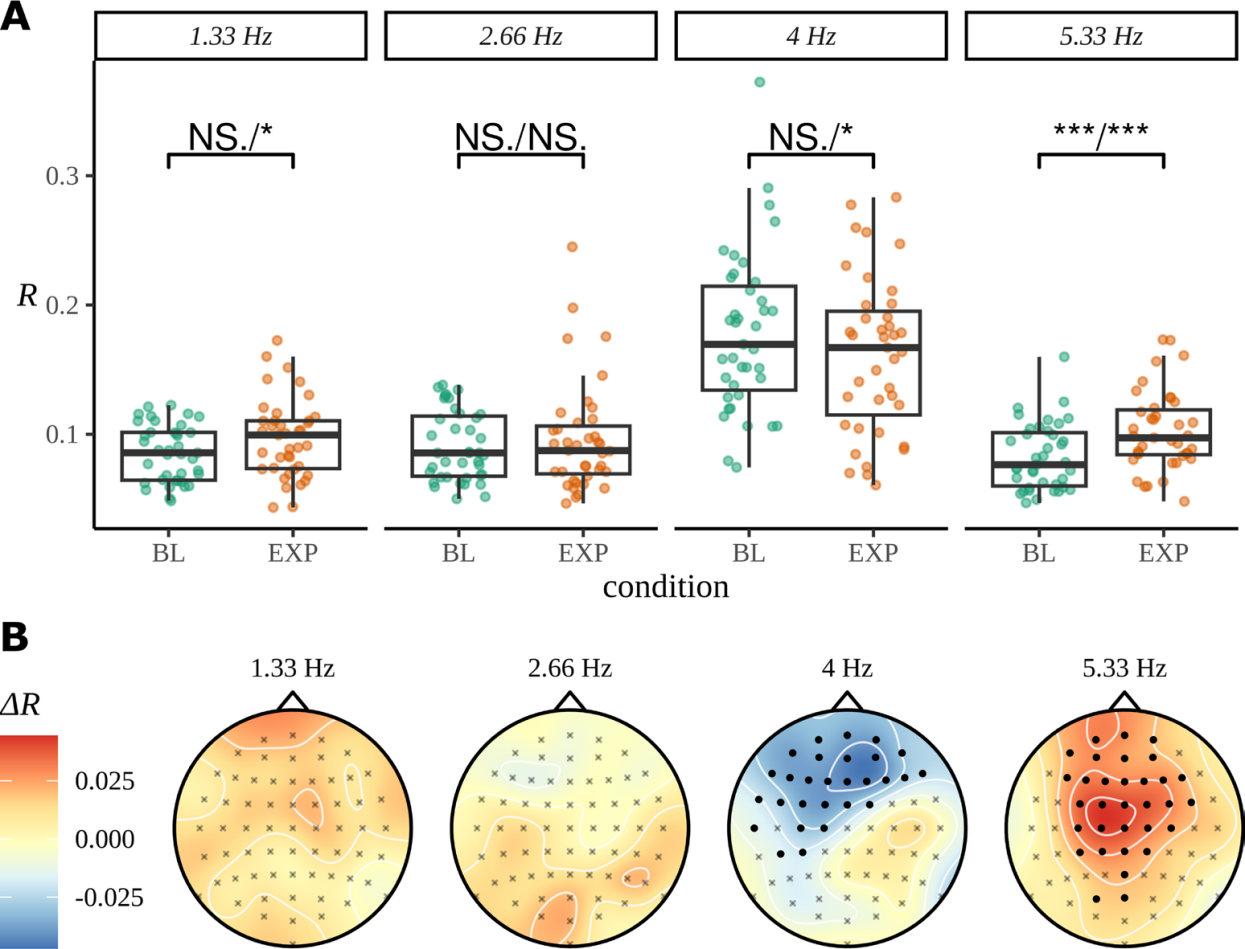
Inter-trial phase coherence (ITPC) analysis. (**A**) ITPC averages across all trials and electrodes for each of the 39 participants. We replicate the statistical procedure as stated in Pinto et al., 2022 of paired one-sided test of a greater ITPC mean of experimental condition (EXP) at the pseudoword rate (1.33 Hz) and its first and second harmonics (2.66 Hz, 5.33 Hz). A one-sided test for a larger ITPC value of the baseline condition (BL) at the syllable rate is also performed. Significance values on the specified test results of an uncorrected paired Wilcoxon signed-rank test (left) and an uncorrected paired *t*-test (right). (**B**) Statistically significant clusters of electrodes were found using a cluster-based permutation test between these two conditions at 4 Hz and 5.33 Hz.

As for the phrase data in Figure 2, we perform a standard ITPC analysis at the group level for this dataset. In Figure 10A, we replicate the original statistical analysis in Pinto et al., 2022 with a one-tailed *t*-test for each frequency. Since ITPC is bounded by zero and one, it cannot be normally distributed, so we also present the results of a Wilcoxon signed-rank test. There is a strong response at the syllable frequency (4 Hz) for both the BL and EXP conditions; however, statistical tests give complicated results. A small increase in coherence can be observed at the pseudoword rate (1.33 Hz) and an even stronger one at the second harmonic (5.33 Hz). No significant difference was observed between BL and EXP at the first harmonic (2.66 Hz), although four participants showed a considerable increase in coherence at this frequency, exceeding values 1.5*IQR above the 75th percentile of the data.

The headcaps in Figure 10B present the condition-to-condition difference in ITPC at each electrode averaged across participants (Equation 3) and interpolated across the skull. We used cluster-based permutation testing to identify significant clusters of activity that describe the differences in average electrode response between conditions. No significant clusters of activity were found at the pseudoword frequency or its first harmonic; however, a stronger mid-to-frontal cluster appears at the second harmonic, suggesting a larger ITPC of electrodes in EXP compared to BL. A significant cluster of activity also appears at the syllable rate and describes an opposite effect of condition: frontal electrodes have a larger ITPC for BL compared to EXP.

#### Bayesian analysis

**Figure 11.**
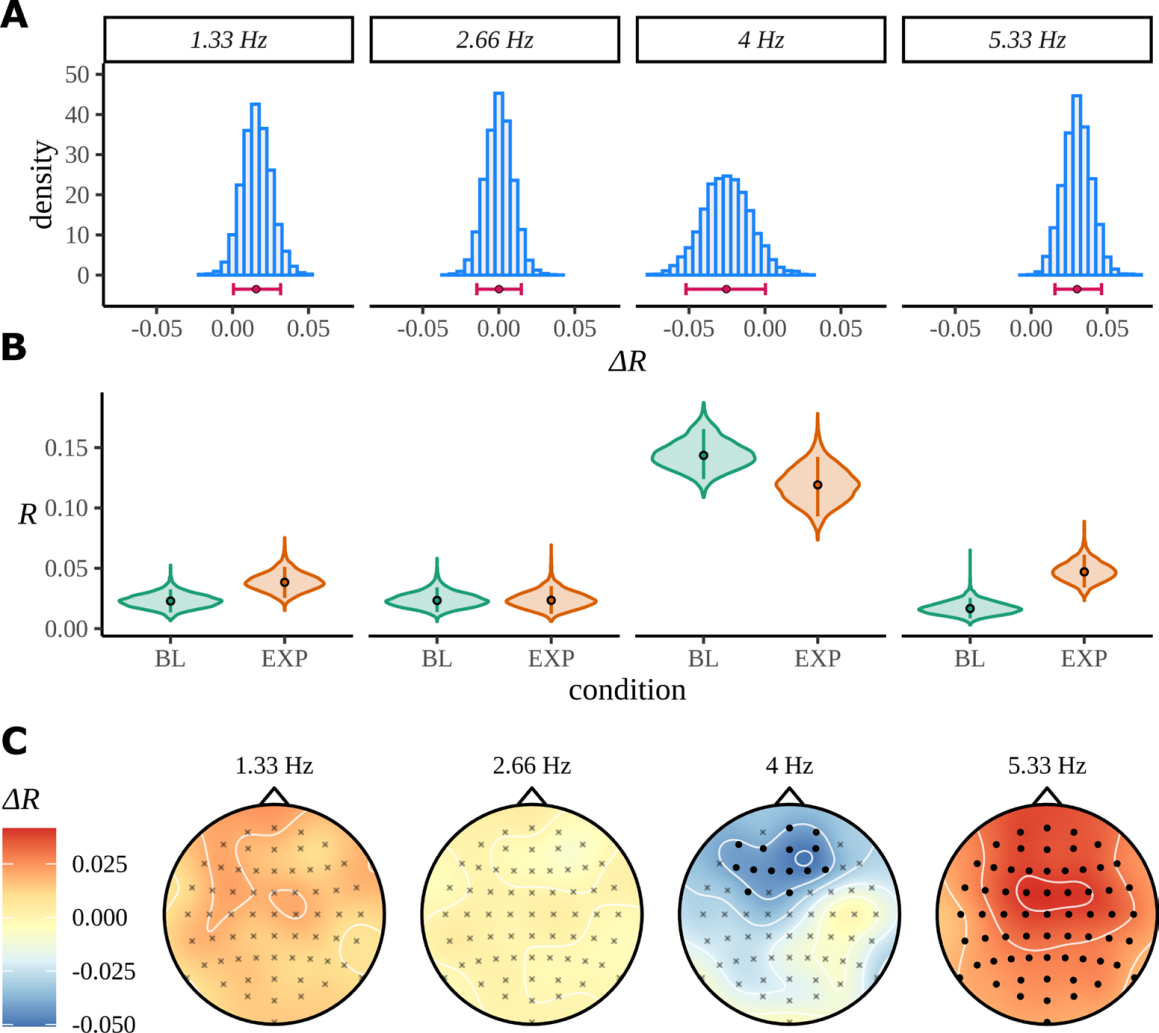
Bayes analysis for the statistical learning data set. (**A**) Posterior distributions over the condition difference EXP-BL are shown for all frequencies of interest. In each case, the full posterior is given by the histogram and is annotated by its 90% highest density interval (HDI) and posterior median. (**B**) Marginal posterior distributions over the mean resultant length for each condition and frequency of interest. (**C**) Posterior means for the difference EXP-BL at each of the 64 electrodes are interpolated across the skull. Filled circles label those electrodes where zero was not contained by the 95% HDI calculated from the quantity in Equation 22.

The model was fit separately for each frequency using four chains sampling for 2000 iterations each, with half of these attributed to the warmup phase. No divergent transitions were detected during sampling, and convergence diagnostic $\hat{R}<1.03$. The posteriors over the difference in mean resultant length for each frequency are shown in Figure 11A. Despite a preference of the posterior at the pseudoword rate to prefer values greater than zero, there are some values in the left tail consistent with no difference. From the summary statistics of the posterior differences in Table 1, we can calculate that zero is approximately 1.6 standard deviations from the posterior mean.

**Table 1.** Summary of posterior values for the statistical learning dataset. All values are rounded to three decimal places. The difference shown is EXP-BL. HDI: highest density interval.

Frequency (Hz)	Mean	Median	SD	90% HDI
1.33	0.016	0.016	0.010	[0.001, 0.032]
2.66	0.000	0.000	0.009	[–0.014, 0.015]
4	–0.025	–0.025	0.016	[–0.052, 0.000]
5.33	0.030	0.030	0.009	[0.016, 0.046]

If we are interested in the full extent of the posterior distribution, it would be more appropriate to calculate the total probability associated with any value of the parameter that supports it. For example, we can calculate from posterior samples that P(ΔR>0|y)≈0.956. This analysis indicates that there is no strong evidence for a large difference in expectation between conditions at the pseudoword frequency, but it is plausible that a difference exists. The first harmonic of the pseudoword rate clearly demonstrates no difference between the experimental groups. The posterior is peaked symmetrically around a mean of zero and has low variance. The second harmonic shows the largest difference between the groups; zero is approximately 3.24 standard deviations away from the mean. Consistent with the ITPC analysis, the results at the syllable frequency also show BL as having a larger value than EXP. In this case, zero lies approximately 1.59 standard deviations from the mean.

Posterior means at each electrode for the same difference are shown in Figure 11B. As before, filled points are those electrodes where the 95% HDI of the marginal posterior over the condition difference does not contain zero. In line with the ITPC analysis for the pseudoword frequency, and its first harmonic (Figure 10B), there is no evidence to suggest any localised patterns of activity occurring in either condition by the Bayesian model. A strong result appears for the second harmonic; according to the Bayesian result, every electrode has a higher mean resultant length in EXP compared to BL. At the syllable rate, a frontal response is discovered; this also reprises the findings from the ITPC analysis.

The Bayesian results give evidence of statistical learning in the second harmonic of the pseudoword frequency; however, results for the pseudoword frequency are not so strong as to rule out no difference entirely. It appears that the strength of conclusions to be made is limited by some participants demonstrating an opposite effect. In Appendix 8, we plot the posterior distribution over the EXP-BL comparison at each frequency and for each participant. In the strongest result, 5.33 Hz, the majority of participants show an increased response in EXP. However in 2.33 Hz and 1.33 Hz, the number of participants that show an opposite effect of condition to those that do not is much more even. In Pinto et al., 2022, it is suggested that evidence of SL is difficult to find in individual participants. The high variance of participant posteriors both within and across frequencies supports this conclusion.

### Simulation study

In this article, we have tried to concentrate on real experimental data: real data often has characteristics and irregularities that are hard to reproduce using fictive data. In our approach here, we have been fortunate that there are datasets which have already been analysed using other, more traditional methods, allowing us to test our method in a situation with something akin to ground truth. Nonetheless, it is useful to also explore the characteristics of our method on fictive data; using fictive data allows us to manipulate effect sizes and the amount of data and contains a ground truth, albeit one located in the perhaps too regular a context provided by simulated data generation.

The Bayesian model reduces bias in the estimation of R. If $R_{i}$ is the true value of the mean resultant length for a condition i, then ${\bar{R}}_{i}$, as calculated by the formula for ITPC (Equation 2), is a positively biased overestimate of this quantity (Kutil, 2012). In a simulation study, we demonstrate that our Bayesian model reduces bias in the estimation of this quantity compared to a typical ITPC analysis. We sampled *R*_1_ and *R*_2_ as ground truth mean resultant lengths for two fictive conditions uniformly over the interval [0,1] by replacing the Beta distribution, which described an explicit prior assumption, with a uniform distribution (see Equation 35). We then use this modified model to generate fictive datasets with different numbers of participants and trials over the sampled ground truth values *R*_1_ and *R*_2_. The estimation bias in each dataset was then analysed with both the ITPC and Bayesian approaches.

**Figure 12.**
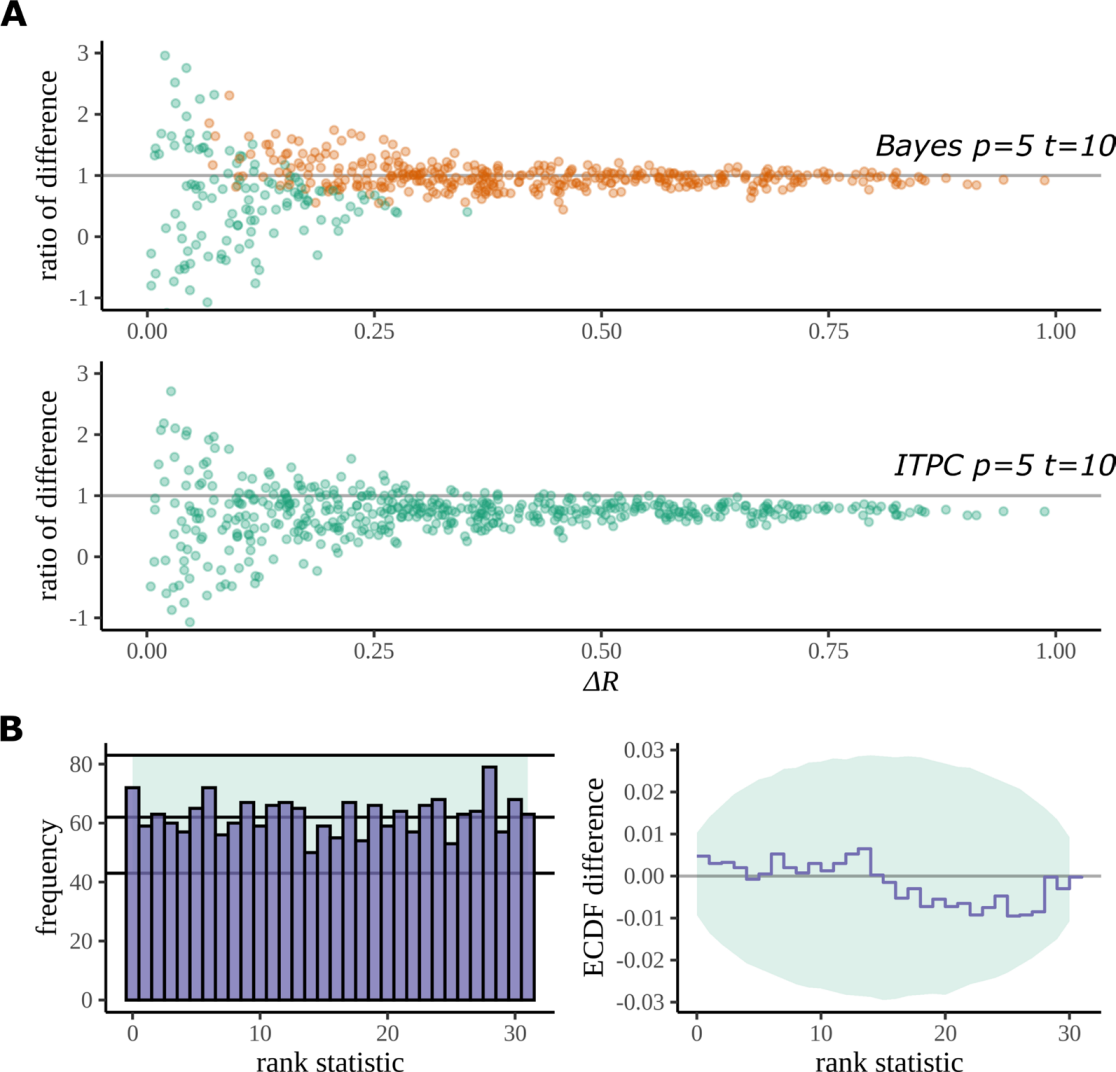
Simulation study. (**A**) The Bayesian model has a higher true detection rate for lower participant numbers. The bias of the estimate is also greatly reduced by the Bayesian model. As the real difference increases along the *x* axis, the variation in model estimates reduces in both methods; however, the distribution of these points around y=1 is much more symmetric for the Bayesian model; this result highlights its bias reduction. The *y* axis has been restricted to help highlight the behaviour of interest. (**B**) Simulation-based calibration for the same participant and trial numbers where the rank of Δ⁢R is analysed. There is no evidence to suggest a tendency of the Bayesian model to overestimate or underestimate the difference.

Figure 12A plots the result of this study for fictive datasets of 5 participants, 10 trials, and 8 electrodes. Each point on the graph is a summary of the performance of each method on its estimation of the true difference in mean resultant length. The x axis gives the absolute value of the true difference, and the y axis gives the ratio of the estimated difference and the true difference:

(24) $$\text{ratio of difference}=\frac{{\bar{R}}_{1}-{\bar{R}}_{2}}{R_{1}-R_{2}}$$

Any systematic deviation from the ideal value of 1 implies a bias in the estimation. Such a trend is present with the ITPC estimation but reduced in the Bayesian one.

Using the same simulated datasets, we looked at how well each method detects a real difference in the mean resultant length. In Figure 12A, points are coloured orange when a difference is correctly detected by the method (zero lies outside the 95% HDI for Bayes, p<0.05 for the ITPC using a paired two-tailed Wilcoxon signed-rank test). The Bayesian model can detect a real difference in mean resultant length for smaller participant numbers. Interestingly, even after a doubling of the number of trials for the same participant number, a difference still cannot be detected by the statistical test: see Appendix 5 and Appendix 6 for the comparison of true-positive and false-positive rates on simulated data between both methods.

We used simulation-based calibration (SBC) (Talts et al., 2018) to demonstrate the calibration of our model. SBC is primarily an algorithm for unit-testing a Bayesian model and is used to provide a visual proof that the model – and its geometry – are not so complex that the sampling algorithm, on average, cannot sample from it without providing a dishonest description of parameters in the posterior. We can use this method to show that our Bayesian model does not provide a biased estimation of Δ⁢R arising from systematic overestimates or underestimates of $\bar{R}$.

Checks for calibration using SBC require that the histogram of rank statistics produced by the algorithm is demonstrably uniform. Figure 12B gives the straightforward histogram of rank statistics and the quantiles containing 99% of the total variation we expect of a true uniform distribution estimated from a finite sample size (details are provided in Appendix 7). The histogram gives no indication of behaviour deviating from a uniform distribution in specific bins or by a more general trend such as a sloping histogram of ranks. Smaller deviations can be hard to detect using this approach, so a second, more sensitive approach is recommended (Talts et al., 2018). The second plot shows the difference between the empirical cumulative distribution function (ECDF) for a uniform distribution and the ECDF estimated from the histogram of rank statistics. The area containing the variability expected of the difference between the uniform ECDF and uniform CDF is shaded. Even with this more sensitive approach no deviation from permissible variation is present. From this result, we can be confident that under its generating process the Bayesian model does not provide a biased estimate of Δ⁢R.

## Discussion

Here, we have presented a Bayesian description of phase data using examples from neurolinguistics. Our approach reprises the original conclusions of the statistical analysis of these data, but, we believe, does so in a more expressive and more natural way. Our account focuses on neurolinguistics, where frequency tagging is common, and we use specific neurolinguistic examples: an example with which we are familiar and for which data is openly available (Burroughs et al., 2021), and an example from a recent study that investigated the presence of statistical learning for an artificial language (Pinto et al., 2022). However, we believe that our approach has broad application across the multiple applications of frequency tagging. Bayesian analysis is, essentially, a more modest approach to data than the more commonly used frequentist analysis: where a frequentist approach seeks to establish with significant certainty whether a hypothesis is true or false, perhaps also using Bayes factors to quantify evidence for a particular hypothesis using a discrete set of models, in a Bayesian analysis we restrict ourselves to the more achievable goal of estimating the values of parameters in a model of the data and calculating our certainty or uncertainty in making those estimates.

### Model extensions

The resemblance of a logistic regression for the scale of the wrapped Cauchy allows the model to be easily adjusted to include other terms typical of a linear model. For example, the statistical learning dataset (Pinto et al., 2022) records from participants in blocks. One extension to the model would be to include an additional term to capture any block effects; alternatively it could also be implemented as an interaction between block and condition.

It is clear from the ITPC headcaps in both analyses that electrodes have spatial correlation. The datasets used in the analyses have been relatively large, both in terms of participants and trials; this has been an important factor in helping the Bayesian model, and the ITPC, resolve activity patterns across the skull. However, for smaller datasets this is not a guarantee. An extension to the model would incorporate prior knowledge about electrode locations, modifying the current independent approach to one that encodes a correlated response between neighbouring electrodes. An initial starting point would be a multivariate normal with a correlation matrix calculated using an appropriate kernel function, such as an exponentiated quadratic, applied to a 2/3D distance matrix of electrode locations.

The statistical learning dataset (Pinto et al., 2022) differs from the phrase dataset (Burroughs et al., 2021) as effects of condition also appear in the harmonics of the main frequency. In the Bayesian analysis we presented, each harmonic is fitted independently of each other. However, if a response is expected at a selection of harmonics of the baseline, then a model that jointly handles these frequencies would be potential avenue for improvement, especially for smaller datasets where information sharing between dependent parameters is a powerful tool for obtaining better estimates.

### Data efficiency

**Figure 13.**
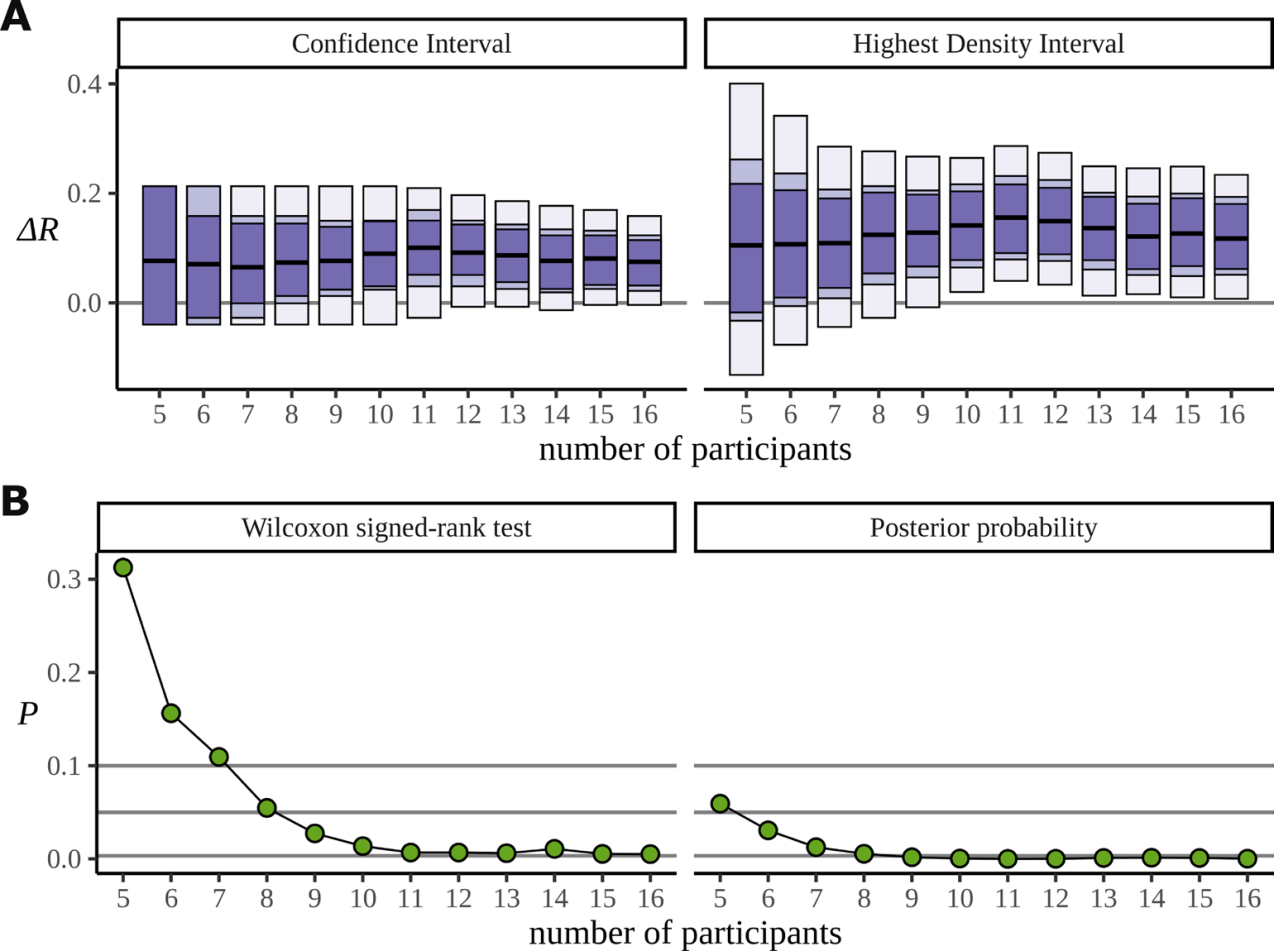
Efficiency of the frequentist and Bayesian approaches for participant number. (**A**) Confidence intervals arising from a two-sided paired Wilcoxon signed-rank (left), alongside Bayesian highest density intervals (right), calculated for the condition difference AN-RR in the phrase dataset. The intervals give widths 90/95/99.7% for each method respectively. (**B**) The p-value arising from the same significance test (left) compared with the probability of observing a value less than zero by the posterior distribution (right).

The Bayesian approach also appears to make more efficient use of the data. In order to investigate the data efficiency of the frequentist and Bayesian approaches, we used the phrase data (Burroughs et al., 2021) and simulated the result we would have had if the experiment had been stopped early, with fewer participants. It can be misleading to directly compare frequentist and Bayesian results; the aims of the two approaches are different. Nonetheless, we have done just that. In Figure 13A, we plot the confidence intervals arising from the two-sided paired Wilcoxon signed-rank test alongside the HDIs from the posterior distribution for decreasing participants numbers. This is produced by removing participants from the data starting with the last recorded. It shows that the posterior still points to a real difference of condition in cases where the low participant number causes the frequentist confidence interval to overlap with zero and fail. The width of these intervals is derived from the critical values outlined below. In Figure 13B, we plot the p-value produced by the same test, and the corresponding probability, calculated from the posterior, of observing a difference less than zero. We also mark the lines for α=0.1, α=0.05, and α=0.003; these correspond to the critical values in an uncorrected one-sided test, an uncorrected two-sided test, and a two-sided test in which a Bonferroni correction of C⁢(6,2) is used to correct for multiple comparisons across the six phrase conditions. We are not advocating for any of these α values, and the uncertainty in deciding an appropriate value of α plagues frequentist approaches. Interestingly, when both participants 15 and 16 are removed from the data, leaving 14 participants, the p-value increases by a factor of approximately 2 (n=15: 0.005, n=14 : 0.011). Figure 7A can explain this result: the posteriors for β show that participant 15 performs better on the task than participant 16, so removing participant 15 from the analysis weakens the result more than removing participant 16.

**Figure 14.**
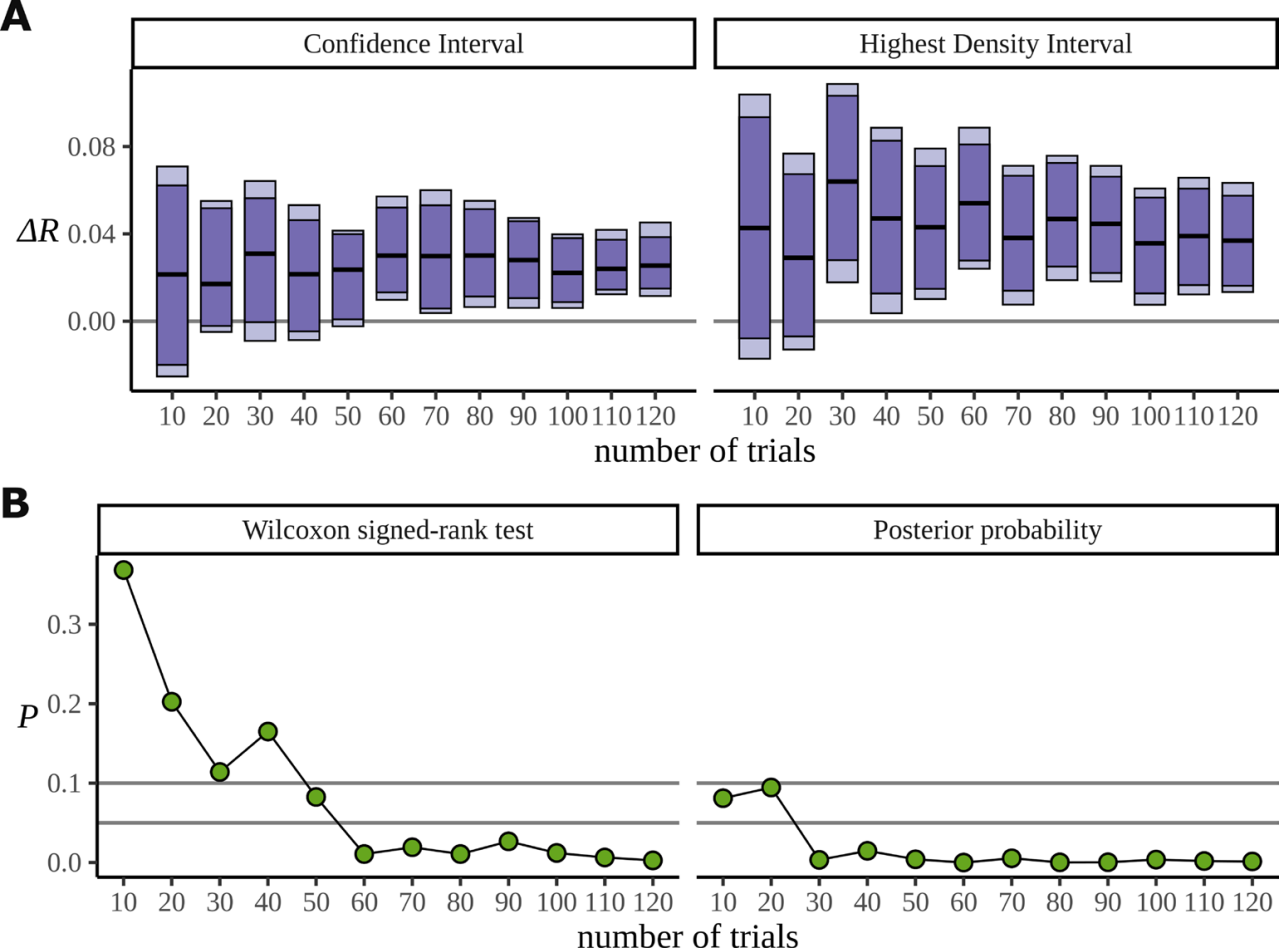
Efficiency of the frequentist and Bayesian approaches for trial number. (**A**) Confidence intervals arising from a two-sided paired Wilcoxon signed-rank (left), alongside Bayesian highest density intervals (HDIs) (right), calculated for the condition difference EXP-BL in the statistical learning dataset. The intervals are given for confidence levels of 90 and 95%. On the right are the HDIs for the same levels. (**B**) The p-value arising from the significance test (left) compared with the probability of observing a value less than zero by the posterior distribution (right).

Figure 14 is similar to Figure 13; however, it uses the statistical learning dataset (Pinto et al., 2022), comparing conditions BL and EXP at the frequency 5.33 Hz. In fact, for these data we saw little evidence that the Bayesian approach works better when the number of participants is reduced: we attribute this to the large number of trials; generally the extra efficiency of a Bayesian approach appears most apparent in low data regimes and the statistical learning dataset is admirably well sampled. For this reason, we used this dataset to investigate data efficiency when the number of trials is reduced for a fixed number of participants. In Figure 14, data from the first 20 participants are considered and the analysis is repeated with different numbers of trials, discarding trials from the end. It is clear from Figure 14A that the Bayesian model can reliably detect the signal in the data with at least half the number of trials that the frequentist approach requires; this is potentially useful especially because of the challenge semantic satiation poses to some neurolinguistic experiments. Figure 14B compares the p-values arising from the significance test with P(ΔR<0) calculated from the posterior and shows the fast convergence of the posterior to the signal; the p-value is much slower and also more variable across trials. For these analyses regarding data efficiency, the degrees of freedom parameter ν was fixed to 30 to address divergent transitions arising for small participant numbers. HDIs were calculated from 8000 posterior samples.

Through simulation we have shown that for lower participant numbers there is evidence that the Bayesian model can detect a true difference more quickly. Similarly, if you have many participants but few trials the Bayesian model also provides a benefit. The probability of making a type 1 error also appeared markedly reduced when using the Bayesian approach for a range of data sizes. Together, these promote the adoption of the Bayesian approach to analysing phase data, especially in studies where data is limited, such as pilot studies, where findings influence the direction of subsequent research.

It may appear that our motivation is contradictory; we first explain that frequency-tagging produces robust encephalography results, but then explain that a new framework is required to analyse these results because they are often too noisy to study using a naïve power analysis. Of course, there is no contradiction; the encephalographic study of cognitive phenomena like language demands both a robust experimental paradigm and a cutting-edge analysis pipeline!

### EEG data can benefit from a Bayesian analysis

The Bayesian approach we have advanced in this article is undoubtedly much more computationally demanding than a frequentist approach; it also demands some thought and experiment in the formulation of the model and its priors. Frequency tagging is, in this regard, a particularly demanding application of the approach. However, we believe that the clarity of a Bayesian description and the complete way it presents the model and its evidence, along with the great data efficiency it provides, make it superior. Some of the complexity of our approach derives from the difficulty of sampling a circle, and we hope this example will be helpful in incorporating compact distributions into the standard probabilistic packages such as Stan and Turing.

In general, Bayesian models become worth the effort in scenarios with two properties: (1) where the data are limited and noisy, so statistical uncertainty is high and therefore worth representing explicitly; (2) where the dataset has a strong structure, which the Bayesian model can be designed to match and therefore share information across parameters. For these reasons, we also believe that similar Bayesian approaches will have broad application to EEG data. The nature of EEG data, its noisiness high-dimension, and the tendency to small participant numbers make it likely that Bayesian methods will be helpful. This certainly is evident in the preliminary work report in Turco and Houghton, 2022.

## Figures and Tables

**Appendix 5—figure 1.**
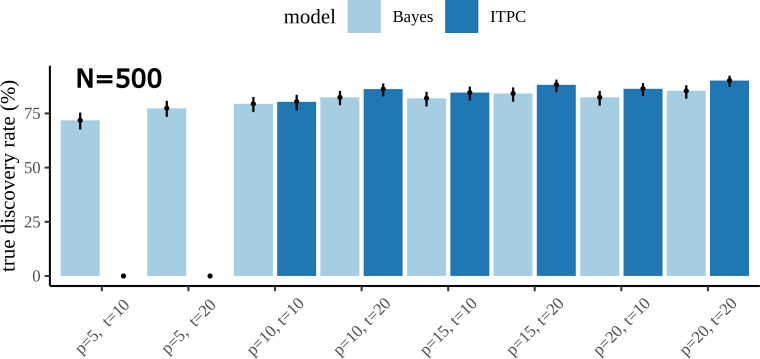
True discovery rates. A summary of more participant and trial numbers of the plots shown in Figure 12A. For five participants, a doubling of the number of trials still provides insufficient information for the inter-trial phase coherence (ITPC) and resulting significance test to conclude that a difference exists in any simulation. Bars show the 95% confidence intervals on this measure estimated through bootstrap.

**Appendix 6—figure 1.**
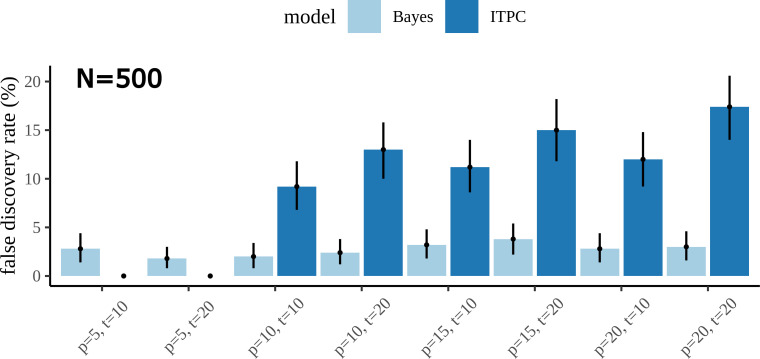
False discovery rates. The frequentist approach to inter-trial phase coherence (ITPC) differences using a paired Wilcoxon signed-rank test has a type 1 error that increases with both participant and trial number. Bars show the 95% confidence intervals on this measure estimated through bootstrap.

**Appendix 8—figure 1.**
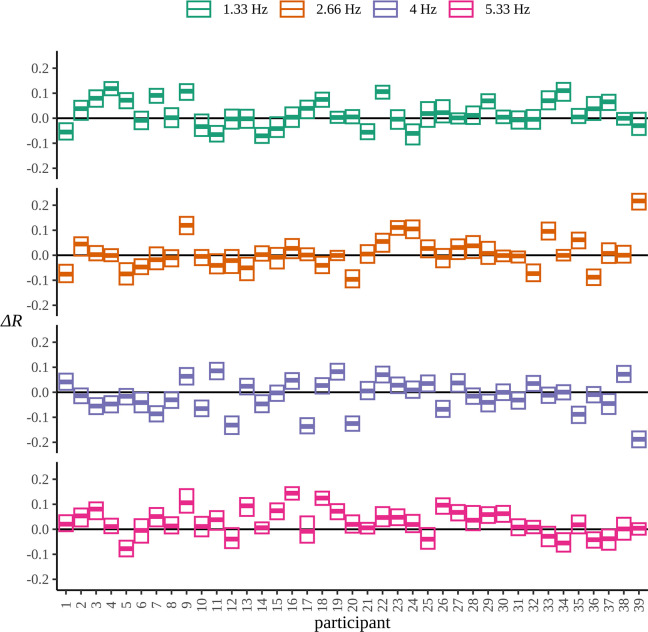
Participant posteriors. Highest density intervals containing 99% of the posterior probability for each participant and frequency over the difference in mean resultant length EXP-BL.

**Appendix 9—figure 1.**
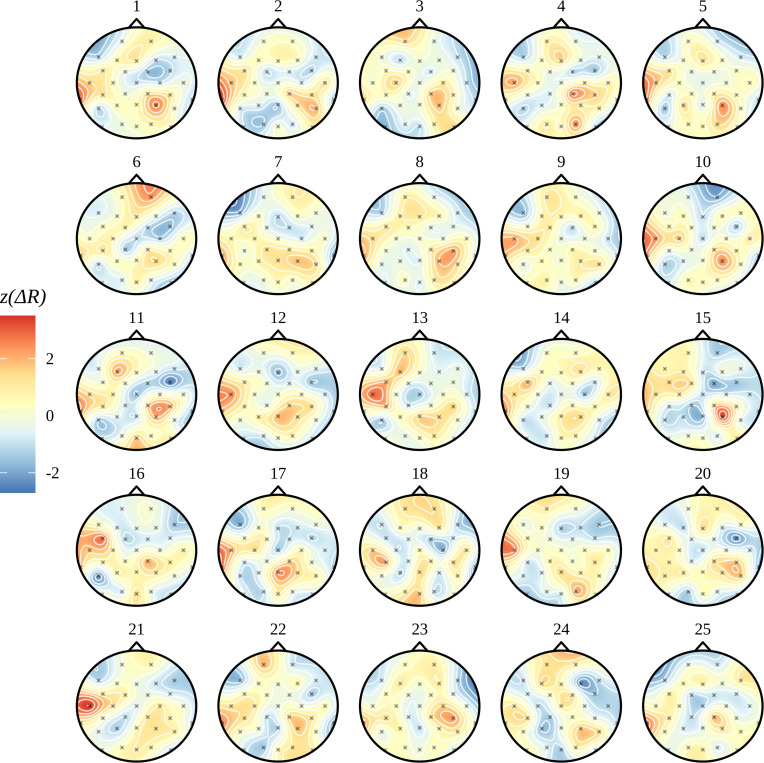
Joint headcap posterior. Here we visualise uncertainty in the posterior over the difference AN-AV and how it captures a range of plausible activity patterns. As expected, samples demonstrate variation about the mean shown in Figure 6B of a right parietal and left temporal activation. AN: adjective–noun; AV: adjective–verb.

## Data Availability

This manuscript is a computational study, so no data have been generated. All modelling code for this study is available from GitHub (also provided in appendix 2). The statistical learning dataset used as a case study in this paper is available from OSF (contact author EZ Golumbic for data related correspondence). The following previously published datasets were used: BurroughsA KazaninaN HoughtonC 2020Grammatical category and the neural processing of phrases - EEG dataZenodo10.5281/zenodo.4385970PMC784429333510230 PintoD GolumbicEZ 2021Assessing the sensitivity of EEG-based frequency-tagging as a metric for statistical learningOpen Science Frameworksyn3h10.1162/nol_a_00061PMC1015857037215560

## Additional files

MDAR checklist

## Appendix 1

### Full model

In the Bayesian model, the individual phases are modelled as draws from a wrapped Cauchy distribution:

(25) $$\theta_{pcek}∼\text{Wrapped-Cauchy}(\mu_{pce},\gamma_{pce})$$

where, as above, p, c, and e are participant, condition, and electrode number, and k is the trial number. The mean phase is derived from the Bundt-gamma distribution:

(26) $$\left(x_{pce},y_{pce})∼Bundt-Gamma(10,0.1\right)$$

The probability density function for the Bundt-gamma distribution can be derived through a Jacobian adjustment from polar to Cartesian coordinates. Our assumptions in polar coordinates are a uniform angle, and a gamma distributed radius:

(27) $$\rho_{pce}∼Gamma(10,0.1)$$

(28) $$\begin{array}{rl} \mu_{pce} & ∼Uniform(-\pi ,\pi ) \end{array}$$

Unlike the choice of a uniform distribution for the mean, the choice for the distribution for the radius is somewhat arbitrary because it has no implication for quantities that we analyse. It is simply a mathematical tool that can promote more efficient sampling by soft-constraining the sampler in parameter space. To represent these assumptions in Cartesian coordinates, we multiply these independent assumptions by the Jacobian of the transformation 1/ρ:

(29) $$p(x_{pce},y_{pce})=\frac{1}{\rho_{pce}}p(\rho_{pce},\mu_{pce})$$

(30) $$\begin{array}{rl} & =\frac{1}{2\pi \rho_{pce}}Gamma(\rho_{pce}|10,0.1) \end{array}$$

This gives an angle uniform on the circle, not on the interval:

(31) $$\mu_{pce}=angle(x_{pce},y_{pce}).$$

As described above, the model for γ uses a pair of link functions so

(32) $$\gamma_{pce}=-\log(1-S_{pce})$$

and

(33) $$S_{pce}=\sigma (\upsilon_{pce})$$

with a linear model for $\upsilon_{p\,c\,e}$:

(34) $$\upsilon_{pce}=\alpha_{c}+\beta_{pc}+\delta_{ce}$$

We have priors for each of $\alpha_{c}$, $\beta_{p\,c}$, and $\delta_{c\,e}$, what in linear regression are referred to as slopes. The prior for $\alpha_{c}$ is induced through placing a prior over $\sigma \,\left(\alpha_{c}\right)$ which represents the baseline circular variance for each condition

(35) $$\sigma \left(\alpha_{c}\right)∼Beta(3,2)$$

By applying the change of variables formula, we can work out the pdf for the prior induced on $\alpha_{c}$:

(36) $$p(a_{c})=Beta(\sigma (a_{c})|3,2)\frac{e^{\alpha_{c}}}{(1+e^{\alpha_{c}}{)}^{2}}$$

As discussed above, for $\beta_{p\,c}$ we have a hierarchical structure modelling covariance of participant responses across conditions, thus:

(37) $$\beta_{p}∼MvT(\nu ,\text{0},\Sigma )$$

where $\beta_{p}$ is a vector over the c index. With *C* conditions, the scale matrix Σ is a C×C matrix. It is made up of a correlation matrix Ω, and a set of scales, $\sigma_{1}$ to $\sigma_{C}$.

(38) $$\Sigma =\text{diag}(\sigma_{1},\ldots ,\sigma_{C})\cdot \Omega \cdot \text{diag}(\sigma_{1},\ldots ,\sigma_{C})$$

To facilitate the interpretation as a covariance matrix, this scale matrix needs to be multiplied by ν/(ν-2). The correlation matrix has a Lewandowski–Kurowicka–Joe prior (Lewandowski et al., 2009; Gelman et al., 1995):

(39) Ω∼LKJ(2.0)

The prior for the degrees of freedom parameter ν is given a gamma prior:

(40) ν∼Gamma(2,10)

and the scales have half-normal priors:

(41) $$\sigma_{c}∼Half-Normal(0,0.5)$$

Finally, for $\delta_{c\,e}$ we partially pool electrodes within condition only:

(42) $$\begin{array}{rl} \delta_{ec} & ∼Normal(0,\tau_{c}) \end{array}$$

(43) $$\begin{array}{rl} \tau_{c} & ∼Half-Normal(0,0.5) \end{array}$$

To attempt a standard notation, we have followed the conventions set by the julia library Distribution.jl by writing the distributions as words and using the same arguments as are found there: in particular, the two parameters for the Gamma distribution correspond to shape and scale.

The prior distributions for β and δ were implemented using a reparameterisation known as *non-centring* (Papaspiliopoulos et al., 2007). This is a commonly adopted technique in hierarchical Bayesian modelling to help alleviate funnels, a class of pathological feature in the target distribution that cause slow and biased sampling. This reparameterisaton does not change the mathematical model; its sole purpose is to help the numerical computation. See McElreath, 2018 and Betancourt and Girolami, 2015 for an introduction to this approach.

## Appendix 2

### Software

Posteriors were sampled using rstan v2.21.5 and cmdstanr v0.5.2. Data and posteriors were analysed using R v4.2.1; tidyverse v1.3.1; reshape2 v1.4.4; and HDInterval v0.2.2. All graphs were plotted in ggplot2 v3.3.6. Figure 2B used ggsignif v0.6.3 for hypothesis testing and additional plotting functionality; Figure 5B used viridis v0.6.2 for heatmap colours; headcaps were interpolated using mgcv v1.8–40 for Figure 2C, Figure 6C, and Figure 7C; ridgeplots were created for Figure 7B with ggridges v0.5.3; Hamiltonian energy distributions were plotted in Figure 9B using bayesplot v1.9.0 (Gabry and Mahr, 2022; Gabry et al., 2019). All panels were composed using inkscape v1.1.1.

### Code and data

The data used here are from the open dataset (Burroughs et al., 2021); all codes are available on GitHub (copy archived at Houghton and Dimmock, 2023).

### Tree depth warnings

The sampler has been observed to produce a low number (<1%) of max_treedepth warnings. This does not imply biased computation like those arising from divergences, but it is a warning about efficiency. A higher tree depth comes at the cost of doubling the number of gradient evaluations required at the previous depth (Hoffman and Gelman, 2014), adding a penalty to the run time.

### Computing resources

Posteriors were sampled locally on a Dell XPS 13 7390 laptop (Intel i7-10510U @ 1.80 GHz, 16 GB of RAM) running under Ubuntu 20.04.4 LTS.

## Appendix 3

### Table of experimental conditions

The six experimental conditions are shown in Appendix 3—table 1.

**Appendix 3—table 1.** Table of conditions for the phrase dataset.

Condition	Description	Example
AN	Adjective–noun pairs	…old rat sad man…
AV	Adjective–verb pairs	…rough give ill tell…
ML	Adjective–pronoun verb–preposition	…old this ask in…
MP	Mixed grammatical phrases	…not full more green…
RV	Random words with every fourth a verb	…his old from think…
RR	Random words	…large out fetch her…

Of these, four are ‘phrase conditions,’ AN, AV, MP, and RR, and were analysed in Burroughs et al., 2021; the other two, ML and RV, are ‘sentence conditions’ which formed part of the experiment and are used to investigate phenomena which proved to be absent. ML stands for ‘mixed lexical’ and provides a four-word analogue of the AV condition, repeating lexical category but avoiding grammatical structure. All stimuli are available; see the data and code availability list in Appendix 2.

## Appendix 4

### Cluster-based permutation testing

For the non-Bayesian results, significant clusters of electrodes were identified using cluster-based permutation testing on the ITPC differences. First the mean resultant length was calculated for each participant, condition, and electrode by averaging over trials at the frequency of interest f.

(44) $$R(f,\phi )=|\frac{1}{K}\sum_{k}e^{i\theta_{fk\phi }}|$$

Cluster-based permutation testing requires a test statistic (separate of any significance testing of clusters) to threshold values, in this case electrodes, that contribute to cluster formulation. The test statistic we chose for deciding if an electrode should appear in a cluster is the mean of the difference in mean resultant length:

(45) $$z_{e}=\frac{1}{P}\sum_{p=1}^{P}\Delta R_{pe}$$

The threshold that signifies an electrode as being important enough to appear in a cluster is based on it being larger than some value that would be unlikely assuming no signal in the data. Specifically, assuming uniformity in the angle of an electrode across trials, and sufficiently large number of trials k≥10, the quantity $R_{p\,e}$ can be modelled using a Rayleigh distribution (Rayleigh, 1880; Horner, 1946).

(46) $$R_{pe}∼Rayleigh(1/\sqrt{2K})$$

To determine a threshold for values of *z*_*e*_ that may be due to a real signal in the data, we bootstrap the distribution of *z*_*e*_ as the mean of the difference of two Rayleigh distributions. This is a distribution of test statistics arising from the assumption that observed phase angles are uniform across trials for each participant, electrode and condition. Values of the 2.5 and 97.5% quantiles of this distribution were used to threshold the test statistic; we estimated

(47) $$\begin{array}{rl} P[z_{e}(P=16,K=24)\leq 0.065] & \approx 0.975 \end{array}$$

(48) $$\begin{array}{rl} P[z_{e}(P=39,K=132)\leq 0.018] & \approx 0.975 \end{array}$$

With this setup defined we can proceed with the cluster-based permutation algorithm. For 5000 iterations, $\Delta \,R_{p\,e}$ was calculated for each participant by randomly swapping condition labels. For this permuted dataset, the value of the test statistic is calculated and electrodes are thresholded. Clusters were formed based on the spatial proximity of thresholded electrodes in a normalised 2D map. The size of the largest cluster for each iteration (sum of the absolute value of test statistics *z*_*e*_ of all electrodes within the cluster) was appended to the null distribution. Finally, this procedure is replicated without permuting the data to calculate a value of the test statistic for the observed data. Clusters identified in the non-shuffled data that had a sum of test statistics greater than the 95th percentile of the approximated null distribution are marked as significant. These appear as filled points in the plots.

## Appendix 5

### True discovery rates

It is important to quantify how well the Bayesian model can correctly detect a true difference in mean resultant length. We simulated from the generative model 500 times for two conditions over a range of participant–trial pairs. From this simulation experiment, we conclude that for lower participant numbers the Bayesian model can detect a true difference much more consistently than the ITPC. The disparity between the two is completely reduced once enough data has been included. From the plot, we can see that the type 2 error is approximately 25% and appears to be slowly decreasing with data size. This is as expected as more data should increase the power of the ITPC, and through a similar reasoning, should also benefit the Bayesian analysis.

## Appendix 6

### False discovery rates

Alongside our comparison of the true discovery rate between the Bayesian model and ITPC, we also looked at the false discovery, or type 1 error rates. This required a slight change in the generative model for the data, namely, setting the true value of the $R_{c}$ in both fictive condition groups equal. Our simulation showed that the false discovery rates between the models are considerably different and dependent on data size. It is immediately clear from the plot below that the Bayesian result outperforms the ITPC in almost every combination of participant and trial number.

To investigate what was driving this discrepancy between the two approaches, we compared the posterior distribution to a bootstrapped sampling distribution of the mean difference for simulated datasets where the Bayesian model gives a true-negative, but the ITPC a false-positive. This highlighted that the Bayesian estimate is a more conservative one, likely taking into account more sources of variation in the data to form its conclusion about the difference. The ITPC was overconfident in its estimates compared to the Bayesian counterpart; its paradoxical trend of increasing false-positive rates with increasing data size is due to making already overconfident conclusions worse. The statistical test only operates on a summary statistic of the data; it does not know about the number of trials, or even the number of electrodes, the Bayesian model does. Increasing participants for the same trial number, over increasing trials for the same, helps reduce the type 1 error rate of the ITPC because this is the only dimension that can effectively inform the test about variation in the population.

## Appendix 7

### Simulation-based calibration

To generate the set of rank statistics, we iterated N=2000 times taking L=1023 post warm up sampled at each iteration. This results in a distribution of 2000 rank statistics: in this case integers in the interval [0, 1023]. Neighbouring ranks were then binned together to reduce the total number of bins down to 32 as necessary to give a trade-off between variance reduction and sensitivity of the histogram (Talts et al., 2018). The horizontal lines on the histogram mark the (0.005, 0.5, 0.995) quantiles from a Binomial distribution that describes the variation expected of counts in any of the 32 rank-bins after N iterations:

(49) Binomial(n=2000,p=1/32)

## Appendix 8

### Participant posteriors

In a frequency-tagged experiment, it may be of interest to the researcher to look for effects of condition in individual participants. From posterior samples, it is possible to construct participant-specific posteriors over the mean resultant length or its difference between condition. In a similar manner to the calculation for electrodes in Equation 22:

(50) $$\Delta R_{p}=R_{c_{1}p}-R_{c_{2}p}$$

where $c_{1}=$ EXP, $c_{2}=$ BL, and

(51) $$R_{cp}=1-\sigma (\alpha_{c}+\beta_{pc})$$

## Appendix 9

### Headcap variation

In Figures 6B and 7C, headcap plots are constructed by interpolating posterior means at each electrode across the skull. This is useful for summarising the result; however, it ignores the joint behaviour of the posterior and how its uncertainty describes a range of similar, but different responses. The plot below shows 25 samples from the AN-AV posterior distribution. Each headcap has been normalised through local z-scoring to prevent large magnitude differences from masking any individual behaviour.
