# Supplementary material for: Bayesian analysis of phase data in EEG and MEG
Source: eLife. 2023 Sep 12;12:e84602. doi: 10.7554/eLife.84602 (PMC10588985; doi:10.7554/eLife.84602)
Supplement: Peer review history

# Editor report: Editor's evaluation

**DOI:** 10.7554/eLife.84602.sa0

**Authors:** Andrea E Martin

**Affiliations:** https://ror.org/00671me87 Max Planck Institute for Psycholinguistics Netherlands

This important work advances the available statistical methods for estimating the degree to which the neural response is phase-locked to a stimulus. It does so by taking a compelling Bayesian approach that leverages the circular nature of the phase readout and demonstrates the added value of the approach in both simulated and empirical datasets.

# Decision letter

**DOI:** 10.7554/eLife.84602.sa1

**Affiliations:** https://ror.org/00671me87 Max Planck Institute for Psycholinguistics Netherlands; https://ror.org/02feahw73 CNRS France

In the interests of transparency, eLife publishes the most substantive revision requests and the accompanying author responses.

**Decision letter after peer review:**

Thank you for submitting your article "Bayesian analysis of phase data in EEG and MEG" for consideration by *eLife*. Your article has been reviewed by 3 peer reviewers, and the evaluation has been overseen by Reviewing Editor Andrea Martin and Senior Editor Joshua Gold. The following individual involved in the review of your submission has agreed to reveal their identity: Benedikt Zoefel (Reviewer #2).

The reviewers have discussed their reviews with one another, and the Reviewing Editor has drafted this to help you prepare a revised submission.

Essential revisions:

1) All reviewers agree that a more extensive quantitative demonstration of the advantages of your methodological approach compared to existing approaches is needed. For example, first, quantifying the advantage of your analysis over the ITPC analysis in the manuscript would be more convincing than the current graphical approach.

Furthermore, it seems that using a *simulation* approach could be helpful. Simulation of common experimental and data situations, as well as extreme or tough cases where traditional methods run into problems (but your method does not, or is more robust), could be persuasive and help make the impact of the approach more demonstrable and quantifiable.

2) Comparison of data – More thorough and extensive quantitative comparison of the performance of your method compared to existing approaches, as all Reviewers mention, could be carried out on multiple (open) datasets of various sample sizes.

3) Reviewer 3 gives helpful concrete suggestions and concerns regarding the impact of this method for statistical inference (viz., mixed models). These, too, need to be addressed, ideally also quantitatively, but could also be addressed formally/mathematically.

4) Reviewer 1 helpfully explains how the perspective of experimentalists needs to be taken into approach in order for the work to have more impact. Similar to (3) above.

*Reviewer #1 (Recommendations for the authors):*

The study by Dimmock et al. proposes a Bayesian approach to measuring phase coherence. Although I'm familiar with the kind of EEG data analyzed here, I didn't figure out the purpose of the study. It seems like the aim of the study is neither to provide a more powerful statistical test nor to demonstrate some new neural phenomena. The only purpose seems to provide a Bayesian test, but why do we want it?

If the aim is to provide a more powerful test, it should be compared to classic tests for steady-state responses, such as the ones described in the following article.

Picton, Terence W., et al. "The use of phase in the detection of auditory steady-state responses." Clinical Neurophysiology 112.9 (2001): 1698-1711.

The current article is certainly not written in a way that can be understood by an experimentalist. It doesn't matter too much if the methods are hard to follow, but it does matters if no interpretable results are shown. For example, the authors argue that the topographic plots using the new method have a clearer structure than the traditional ones. As an experimentalist, however, I can't figure out which structure is clearer and why it helps to answer scientific questions.

As a methodological paper, testing the method on multiple datasets is a basic requirement. More importantly, the method has to have a clear goal and clearly demonstrate how the goal is achieved.

*Reviewer #2 (Recommendations for the authors):*

This paper presents a novel Bayesian approach to testing for phase coherence in neurophysiological recordings. The approach is centred on probability distributions and therefore allows for more fine-grained conclusions about the existence of such phase consistency, in contrast to the often artificial yes/no decision on the acceptance of the alternative hypothesis that can be found in the literature.

I find this manuscript well written and the rationale well explained. The authors demonstrate that their approach can produce similar, but potentially clearer and less noisy results as compared to more commonly applied techniques (such as inter-trial coherence). It remains difficult to quantify differences between the two approaches (Bayesian vs frequentist) – for instance, the authors write that "these graphs [from Bayesian analysis] show a clearer structure than the corresponding ITPC analysis" without providing a quantification of the difference.

Together, this paper will be useful to the community, possibly opening up new ways of analysing phase-locked neural responses.

*Reviewer #3 (Recommendations for the authors):*

This paper proposes a Bayesian take on the inter-trial phase coherence (ITPC) used to estimate how consistent the oscillatory phase is across trials for a given condition of interest. For standard ITPC the statistical power of the comparisons on the group level is determined by the sample size of the dataset since estimates are derived by first averaging across trials to derive a single condition-level estimate per subject. The advantage of the proposed Bayesian approach is that the resulting model is more robust as it is estimated from the trial level without averaging. It also allows us to derive subject-level estimates (slopes) and explore subject-variable noise. The authors illustrated this by replicating the ITPC analysis from the paper by Burroughs et al. (2021a) using the Bayesian ITPC and demonstrating perceivable noise reduction in the resulting estimates across frequencies and topographical EEG plots. Another key advantage of this method, as illustrated by the authors, is the ability to generate stable estimates for much smaller EEG datasets. While the authors show that Bayesian ITPC can replicate the findings obtained with the standard ITPC, it is not clear what advantages the proposed Bayesian approach offers over other previously proposed methods that allow for trial-level modelling such as linear mixed effects modelling. Secondly, a broader and more accessible description of the steps of the model settings, estimation, and the derivation of the summary statistics should be provided to enable the reader to replicate this method for their own dataset

Abstract

1) lines 12-17 please consider re-phrasing as the message here is not very clear. Please be more specific (based on your analysis findings) what Bayesian approach to coherence contributes more than the traditional one? 'More descriptive' and 'data-efficient' are vague descriptions.

Introduction

2) Lines 26-44. Here to help the readers I would recommend communicating your main point early in the paragraph – that measurement of coherence is an important methodological tool in M/EEG research that is used to answer a wide variety of scientific questions, yet there is room for improvement in how ITPC is estimated.

3) Line 84 – 'this plots', instead of 'this graphs'

4) Lines 96-107 – the main message from this section is not clear. Do authors argue that in the per-electrode ITPC approach the Bonferroni correction for multiple comparisons precludes finding meaningful spatial patterns in the data? In such cases, Bonferroni is rarely used, and spatial cluster-based permutation is a typically used approach that is less conservative and allows the finding of significant clusters of spatially connected electrodes.

5) Lines 126-128 – please unpack a bit more what is meant by 'a better description of the data' and 'a narrative phrased in terms of models and their consequence'.

6) Line 161 – here you mean Figure 4?

Methods section

7) Authors provide a detailed explanation and mathematical descriptions for the distributions from which the phase data can be modelled, and parameters are sampled when building up a Bayesian model of the ITPC. The supplementary materials then detail equations behind the full model used. Yet from these two sources of information, it is challenging for the reader to reconstruct the set of steps authors took to derive the results they plot in Figure 5. If the aim of the paper is to have the reader use the Bayesian approach to ITPC for their own datasets a more accessible step-by-step description of the model estimation is necessary – from calculating participant and electrode slopes/estimates to averaging steps used to produce Figure 5. This can be done by expanding relevant sections in the Methods.

8) Other methods such as Linear mixed models that likewise allow trial-level analysis and model subject slopes have been broadly applied to the EEG data and also ITPC. To increase the contribution of this paper, authors need to outline and demonstrate analytically the advantages of the Bayesian approach over these other non-Bayesian methods.

Discussion section

9) The section Model design choices seem to belong in the Results and not the Discussion section.

10) The Data efficiency section is very helpful in demonstrating the advantage of the Bayesian approach for smaller datasets. This section can be expanded by demonstrating further key advantages of the Bayesian approach over other non-Bayesian methods that use a trial-level approach (as proposed in point 8).

[Editors’ note: further revisions were suggested prior to acceptance, as described below.]

Thank you for resubmitting your work entitled "Bayesian analysis of phase data in EEG and MEG" for further consideration by *eLife*. Your revised article has been evaluated by Joshua Gold (Senior Editor) and a Reviewing Editor.

The manuscript has been improved but there are some remaining issues that need to be addressed, as outlined below:

Reviewers 2 and 3 are satisfied with your revisions, however, given that *eLife* works on consensus and the fact that Reviewer 1 is not satisfied with a major concern of theirs from the first round of review, we ask that you directly address Reviewer 1's queries thoroughly. This includes those concerns regarding interpretability for experimentalists, and specifically, that you compare your method to the classic tests for steady-state responses as the Reviewer suggests. Please pay close attention to each of Reviewer 1's queries and address them in full.

*Reviewer #1 (Recommendations for the authors):*

1. The authors did not address my all my comments and I copied them here.

If the aim is to provide a more powerful test, it should be compared to classic tests for steady-state responses, such as the ones described in the following article.

Picton, Terence W., et al. "The use of phase in the detection of auditory steady-state responses." Clinical Neurophysiology 112.9 (2001): 1698-1711.

The current article is certainly not written in a way that can be understood by an experimentalist. It doesn't matter too much if the methods are hard to follow, but it does matter if no interpretable results are shown. For example, the authors argue that the topographic plots using the new method have a clearer structure than the traditional ones. As an experimentalist, however, I can't figure out which structure is clearer and why it helps to answer scientific questions.

2. I'm glad that the authors included a new dataset in the analysis. However, as an experimentalist, I still cannot see why the new method outperforms the traditional ITPC analysis in the newly added experiment. For the session "Case study – statistical learning for an artificial language", we need at least a few conclusions, explicitly stating whether the new method or the traditional method gives a more reasonable result and why.

3. Simulation is also important. However, I can't really understand the "Simulation study" section. What is exactly R1 or R2? Why do we care about the bias? A more helpful simulation is probably just to simulate the time-domain EEG signal (e.g., using sinusoids and noise) and demonstrate that the new method, e.g., can yield statistical significance with fewer subjects.

"We then use this modified model to generate fictive datasets with different numbers of participants and trials", but where are the results? It seems like Figure 11 does not show how the results change with the number of participants and trials.

For the new section on "Data efficiency", why just one dataset and why only 4 participants? Testing two datasets and all possible numbers of participants are minimal requirements. Also, as an experimentalist, I really cannot understand what is shown in Figure 12.

4. "the power is not a useful measure. Instead, the typical approach to frequency-tagged data for cognitive tasks is to use the inter-trial phase coherence." In fact, most of the studies cited in the introduction used power rather than phase analysis.

5. "The Bayesian approach is more descriptive than traditional statistical approaches: it is a generative model of how the data arises and each component is interpretable and informative about data characteristics."

It's great. But why is the method more interpretable? Could you please summarize it in a way that can be understood by experimentalists?

"It is also more data-efficient: it detects stimulus-related differences for smaller participant numbers than the standard approach."

How is this demonstrated in the two datasets? Is there a guideline about how many participants can be saved using the new approach?

# Reply: Author response

**DOI:** 10.7554/eLife.84602.sa2

Essential revisions:

Reviewer #1 (Recommendations for the authors):

The study by Dimmock et al. proposes a Bayesian approach to measuring phase coherence. Although I'm familiar with the kind of EEG data analyzed here, I didn't figure out the purpose of the study. It seems like the aim of the study is neither to provide a more powerful statistical test nor to demonstrate some new neural phenomena. The only purpose seems to provide a Bayesian test, but why do we want it?

If the aim is to provide a more powerful test, it should be compared to classic tests for steady-state responses, such as the ones described in the following article.

Picton, Terence W., et al. "The use of phase in the detection of auditory steady-state responses." Clinical Neurophysiology 112.9 (2001): 1698-1711.

The current article is certainly not written in a way that can be understood by an experimentalist. It doesn't matter too much if the methods are hard to follow, but it does matters if no interpretable results are shown. For example, the authors argue that the topographic plots using the new method have a clearer structure than the traditional ones. As an experimentalist, however, I can't figure out which structure is clearer and why it helps to answer scientific questions.

As a methodological paper, testing the method on multiple datasets is a basic requirement. More importantly, the method has to have a clear goal and clearly demonstrate how the goal is achieved.

We agree with this comment: our paper presents a novel approach to the analysis of phase data and so should be tested on additional datasets. Stress-testing a novel method is paramount to finding where it excels and perhaps where it does not, and is an necessary step to both increase confidence in the method and promote its adoption.

In our improved manuscript we have provided a detailed analysis of another dataset from a frequency tagged experiment that investigated the role of statistical learning in an artificial language (Pinto et al., 2022). As with the first dataset (Burroughs et al., 2021a, 2021b), we use our Bayesian model to compute statistics equivalent of the frequentist approach and compare the results. Please see: Materials and methods *>* Data for a description of this new data, and Case study – statistical learning for an artificial language, for the results.

Additionally, we analysed the performance of our Bayesian model through a simulation study. This compared the two approaches on the following quantities:

– reported discovery of a difference when it exists (true-positive)

– reported discovery of a difference when it does not exist (false-positive)

We found that the Bayesian model can not only detect a true difference in mean resultant length between conditions with fewer data than the ITPC approach, it also has a greatly reduced false positive rate. These results are described in Simulation study, and appendices 5-6.

As a consequence of this simulation study we also discovered that our Bayesian model reduces bias in the estimation of mean resultant length. The value as calculated in the ITPC analysis is positively biased (Kutil, 2012), however, as demonstrated by our simulation study the Bayesian estimates do not show the same systematic bias as the ITPC. To provide some more rigour to this claim we also demonstrate through simulation based calibration (Talts et al., 2018), evidence of a non-biased estimation by the Bayesan model.

Reviewer #2 (Recommendations for the authors):

This paper presents a novel Bayesian approach to testing for phase coherence in neurophysiological recordings. The approach is centred on probability distributions and therefore allows for more fine-grained conclusions about the existence of such phase consistency, in contrast to the often artificial yes/no decision on the acceptance of the alternative hypothesis that can be found in the literature.

I find this manuscript well written and the rationale well explained. The authors demonstrate that their approach can produce similar, but potentially clearer and less noisy results as compared to more commonly applied techniques (such as inter-trial coherence). It remains difficult to quantify differences between the two approaches (Bayesian vs frequentist) – for instance, the authors write that "these graphs [from Bayesian analysis] show a clearer structure than the corresponding ITPC analysis" without providing a quantification of the difference.

Together, this paper will be useful to the community, possibly opening up new ways of analysing phase-locked neural responses.

Thank you for this encouraging review of our paper. As you have pointed out, it can be difficult to compare Bayesian and frequentist results, and this is certainly something we experienced throughout this project. Instead of strict one-to-one comparisons, that may not always be possible and which, in any case would be against the spirit of what a Bayesian analysis attempts to do, we pursued a qualitative approach, and compare the methods based on the description of the data they provide in their conclusions. For example, confidence intervals are not comparable to highest density intervals (HDIs), nevertheless their respective application is similar: to determine an interval and check its support for a hypothesis. We do feel that one of our contributions in our manuscript is to introduce figure types which are analogous to traditional frequentist results figures while sticking to the descriptive rather than hypothesis based spirit of Bayesian inference; examples of this are figures 6A, 10A and 12B.

Reviewer #3 (Recommendations for the authors):

This paper proposes a Bayesian take on the inter-trial phase coherence (ITPC) used to estimate how consistent the oscillatory phase is across trials for a given condition of interest. For standard ITPC the statistical power of the comparisons on the group level is determined by the sample size of the dataset since estimates are derived by first averaging across trials to derive a single condition-level estimate per subject. The advantage of the proposed Bayesian approach is that the resulting model is more robust as it is estimated from the trial level without averaging. It also allows us to derive subject-level estimates (slopes) and explore subject-variable noise. The authors illustrated this by replicating the ITPC analysis from the paper by Burroughs et al. (2021a) using the Bayesian ITPC and demonstrating perceivable noise reduction in the resulting estimates across frequencies and topographical EEG plots. Another key advantage of this method, as illustrated by the authors, is the ability to generate stable estimates for much smaller EEG datasets. While the authors show that Bayesian ITPC can replicate the findings obtained with the standard ITPC, it is not clear what advantages the proposed Bayesian approach offers over other previously proposed methods that allow for trial-level modelling such as linear mixed effects modelling. Secondly, a broader and more accessible description of the steps of the model settings, estimation, and the derivation of the summary statistics should be provided to enable the reader to replicate this method for their own dataset

Thank you for this clear summary of our manuscript. While we are pleased that we have been able to convey the central results, we would like to improve on this by addressing your points.

We appreciate that the communication of the Bayesian model requires extensive mathematical treatment. Due to the nature of the model, the sequence of transformations that are required to change raw values to quantities of interest can become complicated. To help improve clarity, we have provided further detail on how different quantities are related to each other (see point 7 below).

We do not draw a strong distinction between our Bayesian model and Linear mixed models (LMMs). Our Bayesian model is, at its core, a LMM; with fixed effects for condition, and random effects for participants and electrodes. However, the power of the estimation method allows for increased model complexity, such as a custom link function, and a wrapped distribution for the likelihood. For example, bmrs (Bu¨rkner, 2017), is a package based on stan (Carpenter et al., 2017), that aims to provide greater flexibility to multilevel or mixed modelling than maximum likelihood based approaches such as lme4 (Bates et al., 2015), all while using a near-identical syntax for model specification.

Abstract

1) lines 12-17 please consider re-phrasing as the message here is not very clear. Please be more specific (based on your analysis findings) what Bayesian approach to coherence contributes more than the traditional one? 'More descriptive' and 'data-efficient' are vague descriptions.

It is important for us to communicate the proposed benefits of our Bayesian approach clearly and thoroughly. We have expanded on the abstract, taking these points into consideration.

“This Bayesian approach is illustrated using two examples from neurolinguistics and its properties are explored using simulated data. The Bayesian approach is more descriptive than traditional statistical approaches: it is a generative model of how the data arises and each component is interpretable and informative about data characteristics. It is also more data-efficient: it detects stimulus-related differences for smaller participant numbers than the standard approach.”

Introduction

2) Lines 26-44. Here to help the readers I would recommend communicating your main point early in the paragraph – that measurement of coherence is an important methodological tool in M/EEG research that is used to answer a wide variety of scientific questions, yet there is room for improvement in how ITPC is estimated.

Thank you for this recommendation. We agree, the main ideas of this manuscript had not been introduced adequately in the opening paragraphs. We have now changed the text to convey our motivations earlier.

“In an electroencephalography (EEG) or magnetoencephalography (MEG) frequency-tagged experiment the stimuli are presented at a specific frequency and the neural response is quantified at that frequency. This provides a more robust response than the typical event-related potential (ERP) paradigm because the response the brain makes to the stimuli occurs at the predefined stimulus frequency while noise from other frequencies, which will correspond to other cognitive and neurological processes, does not contaminate the response of interest. This provides a more robust response than the typical event-related potential (ERP) paradigm because the response the brain makes to the stimuli occurs at the predefined stimulus frequency while noise from other frequencies, which will correspond to other cognitive and neurological processes, does not contaminate the response of interest. This quantification is often approached by calculating the inter-trial phase coherence (ITPC). Indeed, estimating coherence is an important methodological tool in EEG and MEG research and is used to answer a wide variety of scientific questions. There is, however, scope for improving how the phase coherence is measured by building a Bayesian approach to estimation. This is a per-item analysis which gives a better description of uncertainty. In contrast, the ITPC discards information by averaging across trials. As a demonstration, both approaches are compared by applying them to two different frequency-tagged experimental datasets and through the use of simulated data.”

3) Line 84 – 'this plots', instead of 'this graphs'

Thank you – “This plots the ITPC measure for all six experimental conditions, …”

4) Lines 96-107 – the main message from this section is not clear. Do authors argue that in the per-electrode ITPC approach the Bonferroni correction for multiple comparisons precludes finding meaningful spatial patterns in the data? In such cases, Bonferroni is rarely used, and spatial cluster-based permutation is a typically used approach that is less conservative and allows the finding of significant clusters of spatially connected electrodes.

This was an interesting point, and one that we had overlooked. Bonferroni correcting such a large number of electrodes was a very extreme case, and as you have mentioned, is avoided in practise for an albeit weaker, but fairer cluster-based permutation test (CBPT). This has allowed us to form a more realistic comparison between the Bayesian and ITPC approaches; not a strictly one-to-one comparison, but nevertheless one that invites similar interpretations.

The headcap plots in figure 2B, and figure 9B (for the additional dataset), now include significant clusters of electrodes marked on the skull. The corresponding Bayesian results, figures 6/10, mark electrodes that did not contain zero in their highest density interval. Also, text (lines 119-130) has been added to the manuscript that discusses CBPT, how it applies to the ITPC, and how it compares with the Bayesian results.

5) Lines 126-128 – please unpack a bit more what is meant by 'a better description of the data' and 'a narrative phrased in terms of models and their consequence'.

In light of this comment we have expanded on the text:

“Furthermore, as a Bayesian approach, it supports a better description of the data, by describing a putative abstraction of the stochastic process that may have generated the data while explicitly stating the underpinning assumptions. This replaces a hypothesis-testing and significance-based account with a narrative phrased in terms of models and their consequence so, in place of an often contentious or Procrustean framework based on hypotheses, a Bayesian approach describes a putative model and quantifies the evidence for it.”

6) Line 161 – here you mean Figure 4?

Yes, thank you – Done.

Methods section

7) Authors provide a detailed explanation and mathematical descriptions for the distributions from which the phase data can be modelled, and parameters are sampled when building up a Bayesian model of the ITPC. The supplementary materials then detail equations behind the full model used. Yet from these two sources of information, it is challenging for the reader to reconstruct the set of steps authors took to derive the results they plot in Figure 5. If the aim of the paper is to have the reader use the Bayesian approach to ITPC for their own datasets a more accessible step-by-step description of the model estimation is necessary – from calculating participant and electrode slopes/estimates to averaging steps used to produce Figure 5. This can be done by expanding relevant sections in the Methods.

Thank you for this helpful comment. We agree that it is important for the reader to be clear on the series of steps taken to arrive at the plotted results. To improve clarity, we have added extra equations. Equations 12-13 have been added to show exactly how the circular variance relates to both the mean resultant R, and the scale of the wrapped Cauchy distribution *γ*. Equations 20-21 have been added to the Results section to make more apparent the calculation used to obtain the values plotted in figure 6 (previously figure 5). As an additional example of how one could go about extracting quantities of interest from the Bayesian model we have provided a further example in appendix 8 that shows posteriors over differences in mean resultant length for each participant.

8) Other methods such as Linear mixed models that likewise allow trial-level analysis and model subject slopes have been broadly applied to the EEG data and also ITPC. To increase the contribution of this paper, authors need to outline and demonstrate analytically the advantages of the Bayesian approach over these other non-Bayesian methods.

Our Bayesian model is very similar to a linear mixed model (LMM), we are essentially estimating fixed and random effects corresponding to conditions, participants, and electrodes. However, it affords us much more flexibility than a typical LMM or gLMM. Because of this we felt that in addition to the current frequentist analysis, the paper would not significantly benefit from including a LMM treatment of these data.

While we agree that a LMM may be suitable for modelling the ITPC, we are unaware how one could do this with phase angles, because this requires a simultaneous estimation of the mean resultant R and its circular variance *S*. As we have shown, these require a wrapped distribution. Modelling ITPC values, *R_pce_*, which at the very least have been averaged over trials is a reasonable approach, but restricted compared to our model, both in terms of expressability—no joint model for R and *S*—and data efficiency, by working directly on a summary statistic of the data.

Discussion section

9) The section Model design choices seem to belong in the Results and not the Discussion section.

Yes, we agree. This section has now been moved to Results.

10) The Data efficiency section is very helpful in demonstrating the advantage of the Bayesian approach for smaller datasets. This section can be expanded by demonstrating further key advantages of the Bayesian approach over other non-Bayesian methods that use a trial-level approach (as proposed in point 8).

Providing examples where the Bayesian model outperforms the standard approach that we are comparing it to is important. However, including LMMs into this analysis is out of scope. Instead, to address this point from a different perspective we investigated the benefits of the Bayesian approach compared to the ITPC in a simulation study; this expands upon the data efficiency section to look at bias reduction, false-positive rates, and true-positive rates over various data sizes. We hope that this provides further, interesting, detail to the data-efficiency discussion.

[Editors’ note: what follows is the authors’ response to the second round of review.]

Thank you for resubmitting your work entitled "Bayesian analysis of phase data in EEG and MEG" for further consideration by eLife. Your revised article has been evaluated by Joshua Gold (Senior Editor) and a Reviewing Editor.

The manuscript has been improved but there are some remaining issues that need to be addressed, as outlined below:

Reviewers 2 and 3 are satisfied with your revisions, however, given that eLife works on consensus and the fact that Reviewer 1 is not satisfied with a major concern of theirs from the first round of review, we ask that you directly address Reviewer 1's queries thoroughly. This includes those concerns regarding interpretability for experimentalists, and specifically, that you compare your method to the classic tests for steady-state responses as the Reviewer suggests. Please pay close attention to each of Reviewer 1's queries and address them in full.

Reviewer #1 (Recommendations for the authors):

1. The authors did not address all my comments and I copied them here.

We are sorry we did not address these comments; this was a mistake on our part, the comments in the original reviews which required the most additional research – the addition of a second data set and simulation studies – distracted us from other important comments.

If the aim is to provide a more powerful test, it should be compared to classic tests for steady-state responses, such as the ones described in the following article.

Picton, Terence W., et al. "The use of phase in the detection of auditory steady-state responses." Clinical Neurophysiology 112.9 (2001): 1698-1711.

We have included a discussion of this approach in our introduction; this approach was developed for long stimulation by periodic stimuli recorded using a small number of scalp electrodes, our recordings are shorter, giving poorer frequency resolution, they are less temporally homogenous and are recorded from multiple electrodes; this makes the classic test unsuitable. The classic test are best suited to the classic application of steady-state methods, with simple tone-like stimuli; the hope is that it is becoming possible to apply frequency-tagging to richer, more temporally complex stimuli. We write:

“There are other classical tests of coherence which use phase information. One example is the Rayleigh test [Rayleigh (1880), Rayleigh (1919)], this test can be used to check for either significant departure from uniformity, or from the ‘expected phase’, that is a particular phase angle specified by the researcher based on some other insight into the behaviour. Other test, such as Hotelling’s *T*^2^ apply jointly to phase and amplitude [Hotelling (1931); Picton et al. (1987, 2001)]. These classical approaches are incompatible with the neurolinguistic study presented here. Firstly, it would be difficult to provide an expected phase; as demonstrated in Figure 4, the mean phase angle is highly variable across participants. There is also no substantive prior information available that could be used to supplement this value because language experiments vary from experiment to experiment. Secondly, because of the problem of semantic satiation the experiments we consider here are relatively short an lack the frequency resolution these classical approaches require.”

Beyond these details though, we believe that the Bayesian approach is better because it models the experiment; ultimately classical approaches depend on the skill of the researcher in statistical analysis whereas Bayesian inference will provide a set of methods which will make efficient use of the data by creating a model of a scientific understanding of the process that produces the data. In other words, although these Bayesian approaches are unfamiliar ultimately they will be easier to use for experimentalists! It seems to us that there are lots of intricate non-Bayesian “workarounds” to various data challenges, but in the long run this makes it harder and harder to understand the statistical framework while a Bayesian approach, though unfamiliar, will lead to more straightforward analyses. Even if this turns out not to be the case, it is a possibility that should be explored and we feel we are contributing to that exploration.

The current article is certainly not written in a way that can be understood by an experimentalist. It doesn't matter too much if the methods are hard to follow, but it does matter if no interpretable results are shown. For example, the authors argue that the topographic plots using the new method have a clearer structure than the traditional ones. As an experimentalist, however, I can't figure out which structure is clearer and why it helps to answer scientific questions.

We are very grateful for this comment, we realise we have “under-sold” one of the biggest advantages of our approach: the Bayesian method is less inclined to producing attractive looking but meaningless information, in frequentist statistics there is a danger of false discovery, or if that is corrected for, a risk of losing real evidence in a effort to remove fictive results. We have added a new figure focusing on a comparison of the topographic headmaps, both using the real data and using fictive data. It is clear that the Bayesian approach represents the underlying ground truth better. The new figure is Figure 8 and we write (lines 370 – 388):

“In Figure 2C we see that even for conditions, such as RR and RV, which contain no linguistic structure at the phase rate there are patterns of electrode activity in the topographics headcaps. In contrast, the analogous Bayesian headcaps in Figure 4C did not show similar patterns. We used simulated data to investigate whether the Bayesian model is correctly demonstrating that there is no phrase-level response for these conditions, rather that the other possibility: that the beguiling patterns seen in the ITPC headcaps represent a real activity invisible to the Bayesian analysis. In fact, the evidence points to the first alternative, Figure 8, presents evidence that the Bayesian model is more faithful to the data when there is no meaningful variation in electrode effects. Figure 8A shows the real data again, however, whereas previously the headcap was plotted for differences between conditions, here we fit directly to the RR condition. There is no effect visible for the Bayesian headcap but for the ITPC headcap there are variations that may suggest localised activity, even though this condition does not any structure at the phrase rate. In Figure 8B four datasets were simulated from the generative Bayesian model with electrode effects set to zero, the four simulations are marked as 1-4 in the figure. Except that there is only one condition the simulated data mimics the real data: it has 16 participants, 32 electrodes and 24 trials. These simulations are intended to represent four different iterations of the same experiment, apart from differing in any random numbers they are identical. The data resulting from these four simulations were fitted with both methods. Evidently, the Bayesian results are much closer to the ground truth. The ITPC results show variations that could easily be misinterpreted.”

We are pleased that the new figure so clearly demonstrates that advantage of our approach.

Overall, we have tried to find ways to make a Bayesian analysis interpretable and believe this is a useful contribution on our part, for example, in Figure 6B we introduce a novel Bayesian equivalent to the typical “significance bracket” style of graph.

2. I'm glad that the authors included a new dataset in the analysis. However, as an experimentalist, I still cannot see why the new method outperforms the traditional ITPC analysis in the newly added experiment. For the session "Case study – statistical learning for an artificial language", we need at least a few conclusions, explicitly stating whether the new method or the traditional method gives a more reasonable result and why.

Thank you for this comment. We are pleased that you found the additional dataset useful, but regret that we did not make clear the respective performance of each method on it. To rectify this, we have added a new figure (Figure 14), that compares the two methods in terms of both confidence and highest density intervals, and *p*-values and posterior probability, as a function of the number of trials in the data. This figure shows that although the methods perform similarly on all the data (compare Figure 10 with Figure 11), when considering a lower number of trials the performance of the Bayesian model in detecting the signal in the data is much better: thus, although the original experiment was impressive in the size of its sample and, of course, as a published data set it is an example where the traditional statistical framework produced a result, a smaller experiment would have failed to produce a signal in cases where the Bayesian analysis would have succeeded. We also write (lines 581-594):

“Figure 14 is similar to Figure 13, however, it uses the statistical learning dataset Pinto et al. (2022), comparing conditions BL and EXP at the frequency 5.33Hz. In fact, for these data we saw little evidence that the Bayesian approach works better when the number of participants are reduced: we attribute this to the large number of trials, generally the extra efficiency of a Bayesian approach appears most apparent in low data regimes and the statistical learning data set is admirably well sampled. For this reason we used this data set to investigate data efficiency when the number of trials is reduced for a fixed number of participants. In Figure 14 data from the first 20 participants are considered and the analysis is repeated with different numbers of trials, discarding trials from the end. It is clear from Figure 14A that the Bayesian model can reliably detect the signal in the data with at least half the number of trials that the frequentist approach requires, this is potentially useful especially when because of the challenge semantic satiation posses to some neurolinguistic experiments. Figure 14B compares the *p*-values arising from the significance test with *P*(∆*R <* 0) calculated from the posterior and shows the fast convergence of the posterior to the signal; the *p*-value is much slower and also more variable across trials.”

3. Simulation is also important. However, I can't really understand the "Simulation study" section. What is exactly R1 or R2? Why do we care about the bias? A more helpful simulation is probably just to simulate the time-domain EEG signal (e.g., using sinusoids and noise) and demonstrate that the new method, e.g., can yield statistical significance with fewer subjects.

R1 and R2 refer to the mean resultant lengths of two conditions 1 and 2 in the simulation study; we had failed to say that and have now fixed that silly error.

"We then use this modified model to generate fictive datasets with different numbers of participants and trials", but where are the results? It seems like Figure 11 does not show how the results change with the number of participants and trials.

The results for different participants and trials were given in appendices 6 and 7, however, in light of this point, we felt that structure of this section was not helpful. It has now been re-arranged to flow in a more obvious way, and the importance of appendices 6 and 7 to the simulation results has been made clearer in the text (lines 467-476).

For the new section on "Data efficiency", why just one dataset and why only 4 participants? Testing two datasets and all possible numbers of participants are minimal requirements. Also, as an experimentalist, I really cannot understand what is shown in Figure 12.

We go to a minimum of five participants to accommodate the parameters in the Bayesian model that estimate participant variance. With too little these parameters will not be informed. Similarly this is why a certain number of groups are recommended for random effects models, as it is important to obtain a reasonable estimate of their variance.

Our previous Figure 12 (now adapted into Figure 13) aims to show that the Bayesian model can detect a difference between conditions using fewer participants. We compare confidence intervals of widths 90/95/0.996 to Bayesian highest density intervals of the same. Depending on how conservative the analyst is about their significance level *α*, small to large differences arise between the methods in terms of the number of participants they require to convincingly detect the signal in the data.

4. "the power is not a useful measure. Instead, the typical approach to frequency-tagged data for cognitive tasks is to use the inter-trial phase coherence." In fact, most of the studies cited in the introduction used power rather than phase analysis.

That is a good point; while power is frequently sufficient for some tasks, the ITPC becomes necessary for more difficult cognitive tasks, for example, power is not used in neurolinguistics since it rarely succeeds in identifying a signal. We have adjusted our text to reflect this:

”the induced power [...] does not work, empirically this proves too noisy a quantity for the stimulus-dependent signal to be easily detected and, indeed, although the frequency tag produces a more robust signal than an ERP, for more high-level or cognitive tasks, particularly neurolinguistic tasks, where frequency-tagging is now proving valuable, the power is not a useful measure; more needs to be done to remove the noise. Typically this is done by assuming the response is phase locked to the stimulus and so for frequency-tagged data in cognitive tasks it is common to use the inter-trial phase coherence.”

5. "The Bayesian approach is more descriptive than traditional statistical approaches: it is a generative model of how the data arises and each component is interpretable and informative about data characteristics."

It's great. But why is the method more interpretable? Could you please summarize it in a way that can be understood by experimentalists?

We don’t believe the current approaches are easy to interpret; the issue of how to correct for multiple comparisons is very fraught for example, and there is no real clarity as to what the appropriate correction is. In the case of ITPC there is a lot of different standards used in claiming a response is significant, based on comparison with simulated data, or data at other nearby frequencies; it is very hard to decide what the correct approach is or how to interpret results that appear significant using one standard and not for another. As another ITPC example, often, two conditions are compared by t-test even though, since ITPC is bound by zero and one, the data is not Gaussian; interpreting the Gaussianity of data that passes a test of Gaussianity when it cannot actually be Gaussian is difficult! In contrast, a Bayesian analysis makes a more modest claim, it gives a description of a model and estimates the posterior of the model parameters based on the data. The familiarity we feel for the traditional methods are the result of years of exposure, we cannot hope to make the Bayesian approach equally familiar to experimentalists in a single paper, but we hope our use of multiple figures, two real data sets and two simulated data sets will be a step towards that goal.

"It is also more data-efficient: it detects stimulus-related differences for smaller participant numbers than the standard approach."

How is this demonstrated in the two datasets? Is there a guideline about how many participants can be saved using the new approach?

We demonstrate the data efficiency of the Bayesian model on the two datasets in Figure 13 and Figure 14 respectively. Figure 13 shows that the number of participants can be reduced when using the Bayesian model, the amount depending on how cautious the analyst wants to be regarding false positives. Figure 14 is a new addition that looks at this problem as a function of trials instead of participants. Here it is shown that the Bayesian model detects the signal in the data with approximately half the number of trials required by the ITPC. Please also see our response to comment 2, and 3b.
